# Analysis of Volatile and Nonvolatile Constituents in Gin by Direct-Infusion Ultrahigh-Resolution ESI/APPI FT-ICR Mass Spectrometry

**DOI:** 10.1021/acs.jafc.3c00707

**Published:** 2023-04-27

**Authors:** Yanning Dou, Marko Mäkinen, Janne Jänis

**Affiliations:** Department of Chemistry, University of Eastern Finland, P.O. Box 111, FI-80101 Joensuu, Finland

**Keywords:** gin, alcoholic beverage, high-resolution mass spectrometry, FT-ICR, foodomics

## Abstract

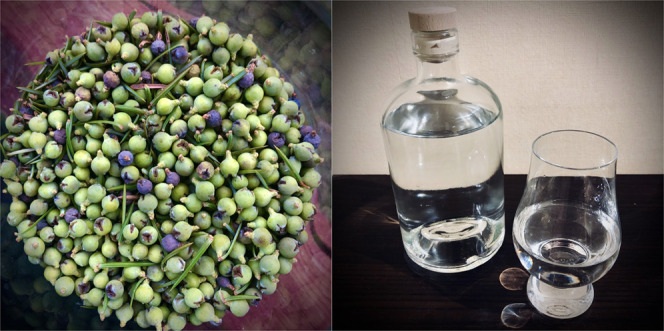

Gin is one of the most consumed distilled alcoholic spirits worldwide, with more than 400 million liters sold every year. It is most often produced through redistillation of agricultural ethanol in the presence of botanicals, most notably juniper berries, which give gin its characteristic flavor. Due to its natural ingredients, gin is a complex mixture of hundreds of volatile and nonvolatile chemical constituents. In this work, ultrahigh-resolution Fourier transform ion cyclotron resonance (FT-ICR) mass spectrometry was used for the compositional analysis of 16 commercially produced gins. Two complementary ionization methods, namely, electrospray ionization (ESI) and atmospheric-pressure photoionization (APPI), were employed to cover a wider compositional space. Each gin provided unique chemical fingerprints by ESI and APPI, which allowed semiquantitative analysis of 135 tentatively identified compounds, including terpene hydrocarbons, terpenoids, phenolics, fatty acids, aldehydes, and esters. Most of these compounds have not been previously reported in gins. While chemical fingerprints were rather similar between most products, some products contained unique compounds due to their special natural ingredients or the production methods applied. For instance, a barrel-matured gin contained a high content of syringaldehyde and sinapaldehyde, which are typical phenolic aldehydes originated from oak wood. In addition, the relative abundance of vanillin, vanillic acid, gallic acid, coniferyl aldehyde, and syringaldehyde was clearly higher than in the other gin samples. Ultrahigh-resolution FT-ICR MS serves as a powerful tool for direct chemical fingerprinting of gin or any other distilled spirit, which can be used for rapid product quality screening, product optimization, or possible counterfeit product discovery.

## 1 Introduction

Gin is an alcoholic beverage famous for its unique taste, differing considerably from the other common spirits. About 800 million liters of gin was sold in 2021, making it one of the most consumed distilled spirits worldwide. There are several ways of producing gin; however, redistillation of grain-based ethanol in the presence of various natural botanicals is the most traditional one. Legally, “distilled gin” is defined by the European Union Regulation (EU) 2019/787 as “a juniper-flavored spirit drink, produced exclusively by distilling ethyl alcohol of agricultural origin,” and has minimum alcohol strength of 37.5% by volume (ABV).^1^

The main flavor of traditional gin originates from juniper (*Juniperus communisL*.) berries.^2^ The other common ingredients include, e.g., coriander, angelica root, cinnamon, liquorice, fruit peels, and/or different berries.^3^ In addition to these ingredients, some gins also have more specific ingredients that make them unique. For example, a Finnish rye-based gin (Kyrö Gin) is based on northern botanicals, including meadowsweet, sea buckthorn, cranberries, and birch leaves. The other rye-based gin from the same distillery (Kyrö Dark Gin) derives its taste from the combination of 17 locally sourced botanicals, and the distillate is matured in the American oak barrels for up to 12 months. These specific ingredients and production methods affect the chemical composition and, thus, the aroma profile of the final product.

Monoterpenes, sesquiterpenes, and diterpenes as well as their derivatives (terpenoids) are the main volatile compounds in gins, mostly originating from juniper berries.^4−6^ These compounds are responsible for the complex taste profiles of many gins. The terpenoid composition characterizes a given gin product and also verifies its authenticity.^7^ Terpenoids are also the main volatile compounds in juniper berries.^3,8^ Monoterpenes (C_10_H_16_) are a family of compounds consisting of two isoprene units. Vichi et al. found 20 different monoterpenes in gins, including α-pinene, β-pinene, limonene, β-myrcene, *p*-cymene, γ-terpinene, and sabinene.^6^ The amount of these compounds considerably varies between different products, ascribing to the citric species in the gin aromatization process.^6^

Oxygenated monoterpenes are another important compound class, contributing to the distinct flavor of gins. Linalool is one of the main oxygenated monoterpenes,^6^ and it is present in many flowers and herbs such as coriander.^9^ Linalool belongs to the class of terpene alcohols, which is important due to its naturally pleasant odor and its antibacterial properties.^10^ α-Terpineol and geranyl acetate are also among the major oxygenated monoterpenes in gins.^6^ α-Terpineol is a monoterpene alcohol which can be found in many plants.^11,12^ Geranyl acetate is an ester of geraniol and acetic acid, and it is used as a flavoring agent or in perfumes.^6^

Sesquiterpenes (C_15_H_24_) consist of three isoprene units. They can be modified by rearrangement of C–C bonds or by oxidation to produce corresponding sesquiterpenoids.^13^ The main sesquiterpenes found in gins are γ-cadinene, δ-cadinene, caryophyllene, β-elemene, γ-elemene, α-humulene, and germacrene D.^7^ These compounds also exist in juniper berries.^14^ The amount of sesquiterpenes considerably varies between different gins.^6^ Some volatile sesquiterpenoids are also present in juniper berries, but they have not been detected in gins.^3^ So far, all of the identified oxygenated sesquiterpenes in gins are alcohol derivatives.^15,16^

Diterpenes (C_20_H_32_) consist of four isoprene units, and they include some important biological compounds, such as retinol and retinal (vitamin A and its aldehyde derivative); and phytol,^17^ which shows considerable antimicrobial and anti-inflammatory activity. Some diterpenoids also contribute to the gin aroma,^7^ including three labdane diterpenoids, manool, manoyl oxide, and epi-manoyl oxide, five abietane derivatives, abieta-8,13(15)-dien-18-ol, dehydroabietal, abieta-8,11,13-trien-7-one, *trans*-ferruginol, and 4-epi-dehydroabietol, and two totarane derivatives, *trans*-totarol and *cis*-totarol. Labdane-type diterpenoids have a wide biological activity spectrum (e.g., antibacterial, anti-fungal, or anti-inflammatory activity), and they also possess therapeutic potential against cancer and heart disease.^18^ Abietane-type diterpenoids, such as ferruginol and abietic acid, are typical diterpenoids in gins. Totarol is another labdane diterpenoid, formally a terpenophenolic compound. It possesses high antibacterial activity, thus having a huge potential as a precursor for novel drugs.^19^ It has been found in *Podocarpaceae* (southern hemisphere conifer) and *Cupressaceae* (cypress) species, and it is also present in juniper berries.^20^

Despite being one of the largest-selling alcoholic spirits, the chemical composition of gin has been very scarcely studied.^3,6,7,21−23^ Most studies rely on conventional gas chromatography–mass spectrometry (GC–MS) technique, which is limited to volatile, low-boiling point compounds only. Another important technique to determine the sensory profiles of spirit drinks is gas chromatography–olfactometry (GC–O), which was recently used in combination with GC–MS to find key aroma compounds in two Bavarian gins.^21^ Human sensory evaluation in conjunction with quantitative compositional analysis is the most important combination to determine the contributions of individual chemical compounds to the overall aroma profile of a given product. However, nonvolatile (polar) constituents in gin, originating from natural ingredients, have been poorly characterized, despite their apparent contribution to the distinct aroma profiles of different gins.

Fourier transform ion cyclotron resonance mass spectrometry (FT-ICR MS) is an ultrahigh-resolution mass spectrometry technique that enables the analysis of complex organic mixtures directly without chromatographic separation.^24^ When compared to conventional GC–MS or liquid chromatography–mass spectrometry (LC–MS)-based approaches, FT-ICR MS allows nontargeted analysis of thousands of chemical constituents in a given sample in just a few minutes. Moreover, when combined with different ionization techniques, like electrospray ionization (ESI) or atmospheric-pressure photoionization (APPI), both polar and nonpolar compounds can be targeted. This technique has been successfully used in the past for direct chemical fingerprinting of different alcoholic beverages like whiskey, rum, wine, and beer.^25−32^ Here, nontargeted chemical fingerprinting of 16 commercial gins was performed by using a direct-infusion ultrahigh-resolution FT-ICR mass spectrometry with negative-ion (−) ESI and positive-ion (+) APPI. The main aim of this work was to assess the suitability of direct-infusion ESI/APPI FT-ICR MS for rapid chemical profiling of gin, both its advantages and possible limitations, and to target especially the nonvolatile (polar) compounds present in the gin samples.

## 2 Materials and Methods

### 2.1 Gin Samples

Table 1 shows the list of 16 gins studied in this work. All of the gin samples were obtained from commercial sources or directly from the gin distilleries. The studied products included mainly grain-based gins (comprising three rye-based Finnish gins) and one wine distillate-based gin.

**Table 1:** List of 16 Commercial Gins Studied in This Work

code	brand name (previous name)	distiller	country of origin	alcohol content (% ABV)	notable ingredient botanicals (other than juniper berries)/other notes
G1	Arctic Blue Gin	Nordic Premium Beverages	Finland	46.2	spruce needles, bilberry leaves, cardamom
G2	Bombay Sapphire	Bombay Spirits Company	U.K.	40.0	ten different botanicals
G3	Roku Gin	Suntory	Japan	43.0	six local botanicals, separately distilled
G4	Filliers Dry Gin 28	Filliers Distillery	Belgium	46.0	28 different botanicals (e.g., hops, angelica roots)
G5	Gaigin	Helsinki Distilling Company	Finland	43.0	yuzu peel, lemongrass, lime, iris, jasmine flower
G6	Helsinki Dry Gin	Helsinki Distilling Company	Finland	47.0	lingonberry, lemon peel, fennel, coriander
G7	Kyrö Helsingin (Helsingin)	Kyrö Distillery	Finland	46.3	pineapple weed (wild chamomile), polypody root; rye-based gin
G8	Hendrick’s Gin	William Grant & Sons	U.K.	41.4	eleven botanicals (e.g., rose petals, cucumber)
G9	Kalevala Gin	Kalevala Distillery	Finland	46.3	mint, rose bud, rosemary, raspberry
G10	Kyrö Dark Gin (Koskue)	Kyrö Distillery	Finland	42.6	17 botanicals; rye-based gin, aged in oak barrels for 3–12 months
G11	Gordon’s London Dry Gin	Tanqueray, Gordon & Co.	U.K.	37.5	coriander, angelica root, licorice
G12	Kyrö Gin (Napue)	Kyrö Distillery	Finland	46.3	17 botanicals (e.g., meadowsweet, birch leaves, cranberry, sea buckthorn); rye-based gin
G13	Nordes Gin	Atlantic Galician Spirits	Spain	40.0	ginger, hibiscus, Albariño grape pomace
G14	Pyy Gin	Teerenpeli Distillery	Finland	45.0	birch leaves, lingonberry, aroma hops
G15	Helsinki Sailor’s Gin	Helsinki Distilling Company	Finland	57.2	lingonberry, lemon peel, fennel, coriander
G16	Xoriguer Mahon Gin	Xoriguer Gin Factory	Spain	38.0	made from distilled wine; oak barrel-matured

### 2.2 Mass Spectrometry Experiments and Data Analysis

All gin samples were analyzed on a 12-T Bruker SolariX XR FT-ICR mass spectrometer (Bruker Daltonics GmbH, Bremen, Germany) equipped with a dynamically harmonized ICR cell (ParaCell), and an Apollo-II atmospheric-pressure ion source, serving both ESI and APPI. For negative-ion ESI, 50 μL of each gin sample was diluted with 950 μL of methanol. The ion source parameters were as follows: capillary voltage +4.5 kV; drying gas temperature 200 °C; drying gas flow rate 4.0 L/min. The samples were directly infused at a flow rate of 5 μL/min using a syringe pump. For positive-ion APPI, 50 μL of each gin sample was diluted with 950 μL of methanol/toluene mixture (9/1; v/v) instead, with toluene serving as a dopant for APPI. Also, the solvent blanks were recorded to identify potential non-analyte signals. The ion source parameters were as follows: capillary voltage −1.5 kV; drying gas temperature 220 °C; drying gas flow rate 4.0 L/min. For both ESI and APPI measurements, 300 time-domain transients (8 MWord) were summed for each spectrum and zero-filled once to obtain the final 16 MWord magnitude-mode data at *m*/*z* 90–1000. The instrument was controlled, and the data were acquired using Bruker ftmsControl 2.1 software. The external mass calibration was done with sodium trifluoroacetate (STFA) clusters prior to the sample measurements.^33^ All of the solvents and reagents were high-performance liquid chromatography (HPLC) grade.

Bruker DataAnalysis 5.0 software was employed for the internal recalibration of the mass spectra using custom-made reference mass lists of commonly observed oxygenated compounds (see the Supporting Information for details). For the peak assignments, the following parameters were used: signal-to-noise ratio (S/N) ≥ 5; relative intensity threshold ≥0.001%; molecular formula: ^12^C_1–100_^1^H_0–300_^16^O_0–30_^14^N_0–2_^32^S_0–1_; maximum number of formulae ≤ 50; double bond equivalent (DBE) ≤ 80; H/C ratio ≤ 3; electron configuration: even (ESI), even/odd (APPI); mSigma ≤ 1000; mass error ≤ 1.0 ppm. The error indicated in Tables S7 and S8 is the mean (absolute) mass error for the given compound, averaged over all samples studied. The DBE value of the compound indicates the degree of unsaturation (i.e., number of rings + double bonds) and can be obtained from the equation DBE = *c* – 1/2*h* – 1/2*x* + 1/2*n* + 1 for a compound with the formula of C_*c*_H_*h*_O_*o*_N_*n*_S_*s*_X_*x*_ (X = F, Cl, Br, or I). The data sorting and visualization were done with OriginPro 2018 (OriginLab Corporation, Northampton, MA) and Microsoft Excel 2016 (Microsoft Corporation, Redmond, WA) software. The relative intensities (abundances) for annotated compounds (Tables S1 and S2) were calculated from the absolute ion intensities (monoisotopic ions) so that their sum adds up to 100%.

CompoundCrawler database search engine was used to facilitate structure annotations. In addition, a putative list of the target compounds was generated on the basis of the earlier publications, mainly comprising volatile compounds.^6,22^ Most compound identifications were considered as confidence level 3 or 4 identifications, consistent with the criteria proposed earlier by Schrimpe-Rutledge and co-workers.^34^ Briefly, level 4 identification refers to the unique molecular formula matching with the experimental isotope distribution, while level 3 represents a higher confidence level matching with one or a few putative candidates (i.e., tentative identification).^34^ In some specific cases, identifications could also be considered level 2 (putative structure) if only one structure present in the database matches with the assigned molecular formula; this could be the case with some fatty acids or some other specific structures. To evaluate the data reproducibility, the gin samples G1, G2, and G10 were analyzed five times (technical replicates). These three gins, a Finnish artisan gin (Arctic Blue; G1), a world-famous London dry gin (Bombay Sapphire; G2), and a rye-based barrel-matured Finnish gin (Kyrö Dark Gin; G10) were selected to represent a broad field of different types of gins. Principal component analysis (PCA) of the data was performed with OriginPro 2018 software, as explained previously.^27^

## 3 Results and Discussion

### 3.1 Gin Analysis with (−) ESI FT-ICR MS

**Figure 1:**
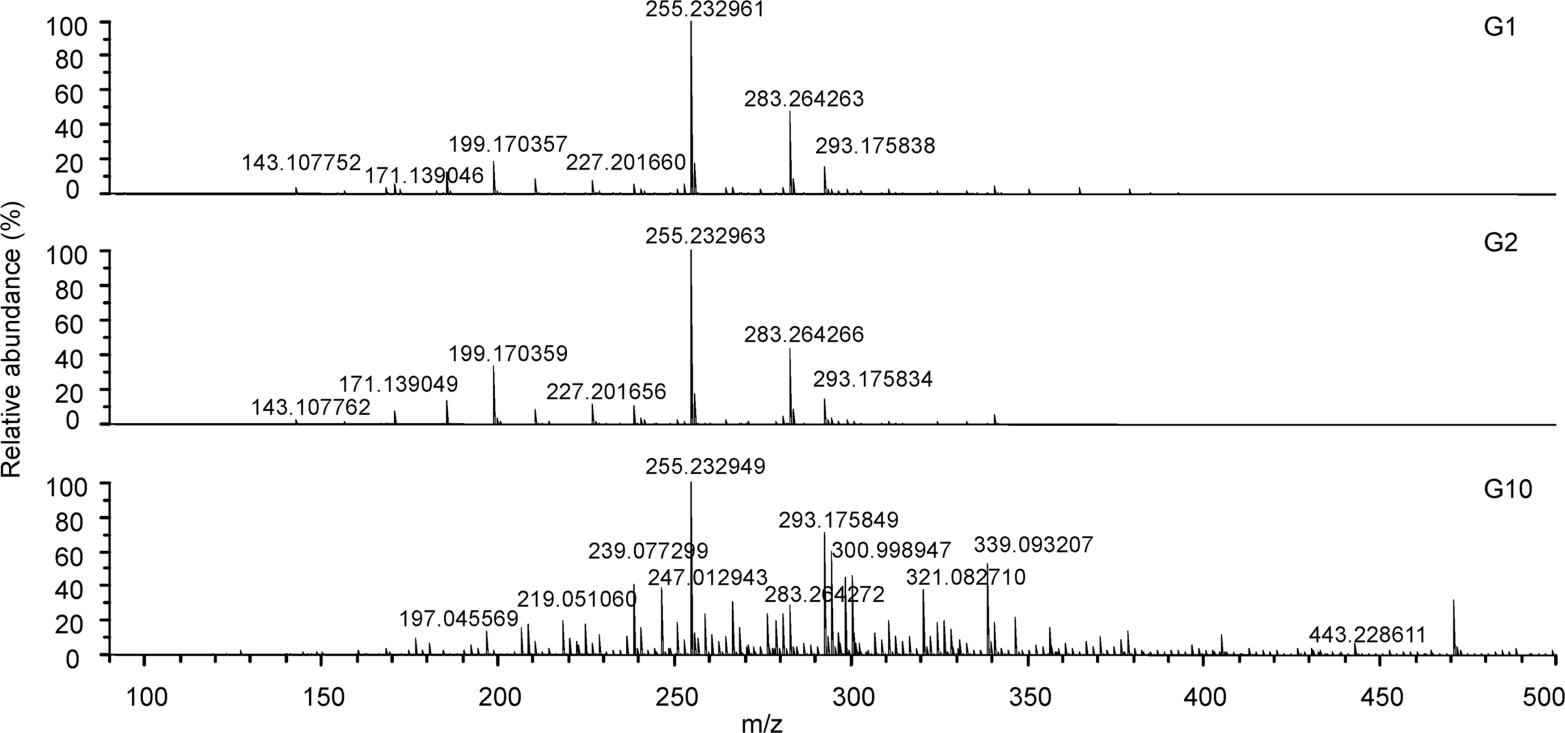
(−) ESI FT-ICR mass spectra of three gin samples.

**Figure 2:**
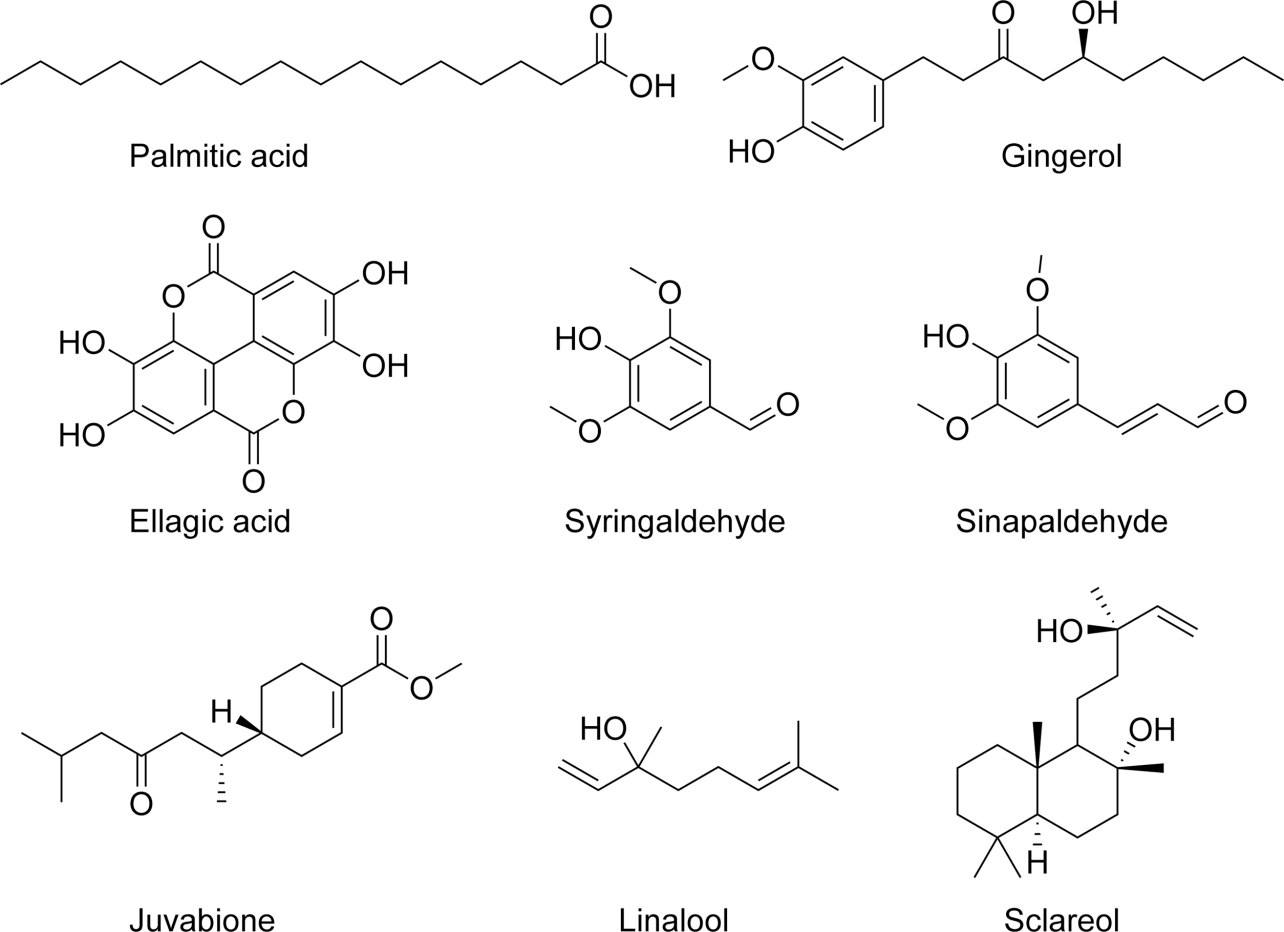
Chemical structures of selected compounds found from gins with (−) ESI.

Electrospray ionization efficiently ionizes polar, heteroatomic compounds. Out of the two polarities, negative-ion ESI preferentially ionizes oxygen-containing compounds and is thus well suited for characterization of alcoholic beverages. Negative-ion ESI FT-ICR mass spectra of three selected gin samples are shown in Figure 1 (for the mass spectra of other gins, see Supporting Information Figures S1 and S2). The most abundant peak observed at *m*/*z* 255.232961 represents palmitic acid, which is a common saturated fatty acid found in many plants. The other abundant compounds across all of the samples studied were stearic acid (*m*/*z* 283.264263), lauric acid (*m*/*z* 199.170357), and gingerol (C_17_H_26_O_4_; *m*/*z* 293.175849), a pungent phenolic compound typically found in fresh ginger. The high relative abundance of gingerol in all studied gins suggests that it originates from the juniper berries. However, it has not been previously reported to occur in juniper essential oils. Figure 2 shows structures for eight selected polar, oxygenated compounds found from gins with negative-ion ESI. When dealing with relative intensities or abundances obtained from the mass spectra, one has to be cautious, though. Due to differences in intrinsic ionization efficiencies and ion suppression effects, especially pronounced in ESI, high relative abundance does not necessarily imply high concentration, and vice versa. For example, fatty acids have high ionization efficiencies in negative-ion ESI, and thus may be overrepresented in the data. These effects are less pronounced in APPI in which ionization occurs in the gas phase.

With negative-ion ESI, an average of 1300 unique spectral features (i.e., unique molecular formulae) were detected (Figure S5), when excluding the gin sample G10 (a barrel-matured gin) which showed over 5200 spectral features, mainly due to the presence of oak wood-derived phenolics (see further discussion below).

The ingredients, especially the selection of natural botanicals, used in gin making strongly affect their chemical compositions and, thus, their sensory profiles. In general, the compounds detected in gins with negative-ion ESI were terpenoids (i.e., monoterpenoids, sesquiterpenoids, and diterpenoids), phenolic compounds, lactones, ethers, acids, and trace amounts of some carbohydrates. The Supporting Information data (Table S1) presents 88 tentatively identified compounds in gin samples by (−) ESI FT-ICR MS. Only seven of these compounds have been previously reported. Phenolic compounds and acids were the dominating ones in all of the studied samples due to their high ionization efficiencies. A range of fatty acids (i.e., palmitic, stearic, caprylic, pelargonic, and myristic acid) showed the highest abundance.^35^ In contrast, glucose was the most abundant compound observed in G6 (as the deprotonated molecule, [C_6_H_11_O_6_]^−^, and the chloride adduct, [C_6_H_12_O_6_Cl]^−^). The observation of a high amount of glucose in a distilled spirit is somewhat surprising and must be originating from some of the ingredients of this particular gin. Many phenolic compounds such as, ellagic acid, sinapaldehyde, or ethyl gallate, were much more abundant in G10 as compared to the other gins due to its post-distillation maturation in oak barrels.

The main flavor of many traditional gins originates from juniper berries. Thus, monoterpenes, sesquiterpenes, and diterpenes as well as their oxygenated derivatives are the main compounds detected in most gins.^5,6^ These compounds are mainly responsible for the specific gin aroma profiles, but their concentrations considerably vary between different gin brands. In this study, nine mono-, sesqui-, and diterpenoids were detected by (−) ESI FT-ICR MS, identified as carvone, camphor, linalool, methylionone, spathulenol, elemol, capnellane sesquiterpene, juvabione, and sclareol. One of the main limitations of direct-infusion FT-ICR MS is that structural isomers cannot be directly distinguished. Therefore, these identifications were made by comparing the terpenoid compositions of gins obtained in the earlier GC–MS-based studies, and the most frequently detected compounds were reported. In addition, coriander, angelica, cinnamon, and liquorice are the common ingredients in gins, bringing their own contribution to the aroma profiles.^3^ No specific compounds related to these ingredients could be identified, however.

Barrel aging has been used to bring some additional flavors to gin, similar to other barrel-matured spirits. Koskue (G10) was the only barrel-aged gin analyzed in this study. Koskue has been aged in American oak wood barrels 7–12 weeks, which is readily observable by its yellowish/brownish color. Most phenolic compounds detected, including vanillin, vanillic acid, gallic acid, coniferyl aldehyde, syringaldehyde, ferulic acid, ethyl gallate, and sinapaldehyde, were much more abundant in Koskue than in the other studied gins. Syringaldehyde and sinapaldehyde, for example, are typical phenolic aldehydes derived from oak wood^36^ and give a spicy and smoky aroma to the spirit. Ethyl gallate can be produced by esterification of gallic acid with ethanol.^37^ It is a natural phenolic antioxidant and is used as a food additive. Ellagic acid (C_14_H_6_O_8_; *m*/*z* 300.998947), a polyphenol found in many fruits, was also highly abundant in Koskue but was completely undetectable in most other gins. Ellagic acid can be found in many oak wood species, and it is also found in red wine. Salicylic acid, a phenolic acid found in meadowsweet,^38^ was also detected in Koskue.

### 3.2 Gin Analysis with (+) APPI FT-ICR MS

**Figure 3:**
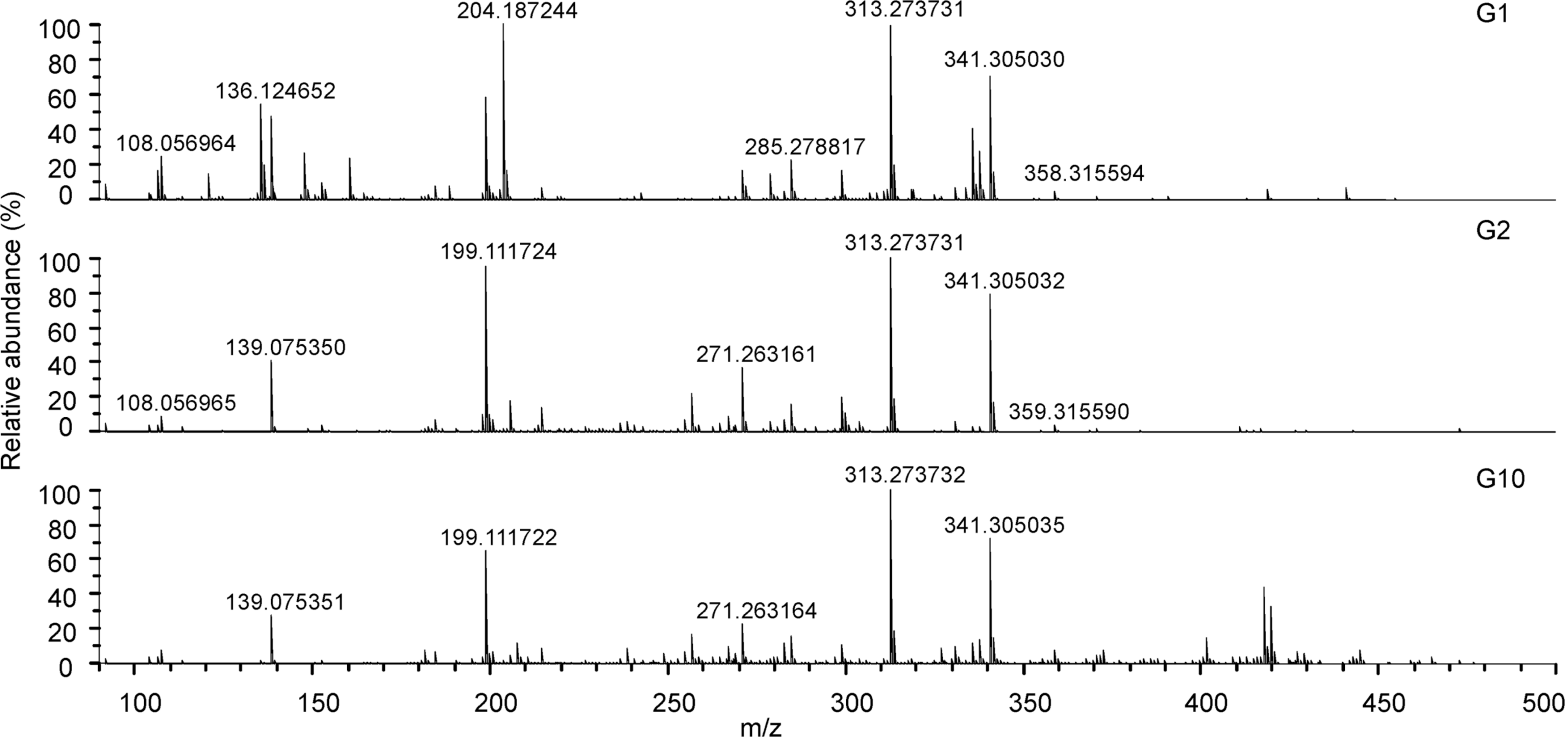
(+) APPI FT-ICR mass spectra of three gin samples.

**Figure 4:**
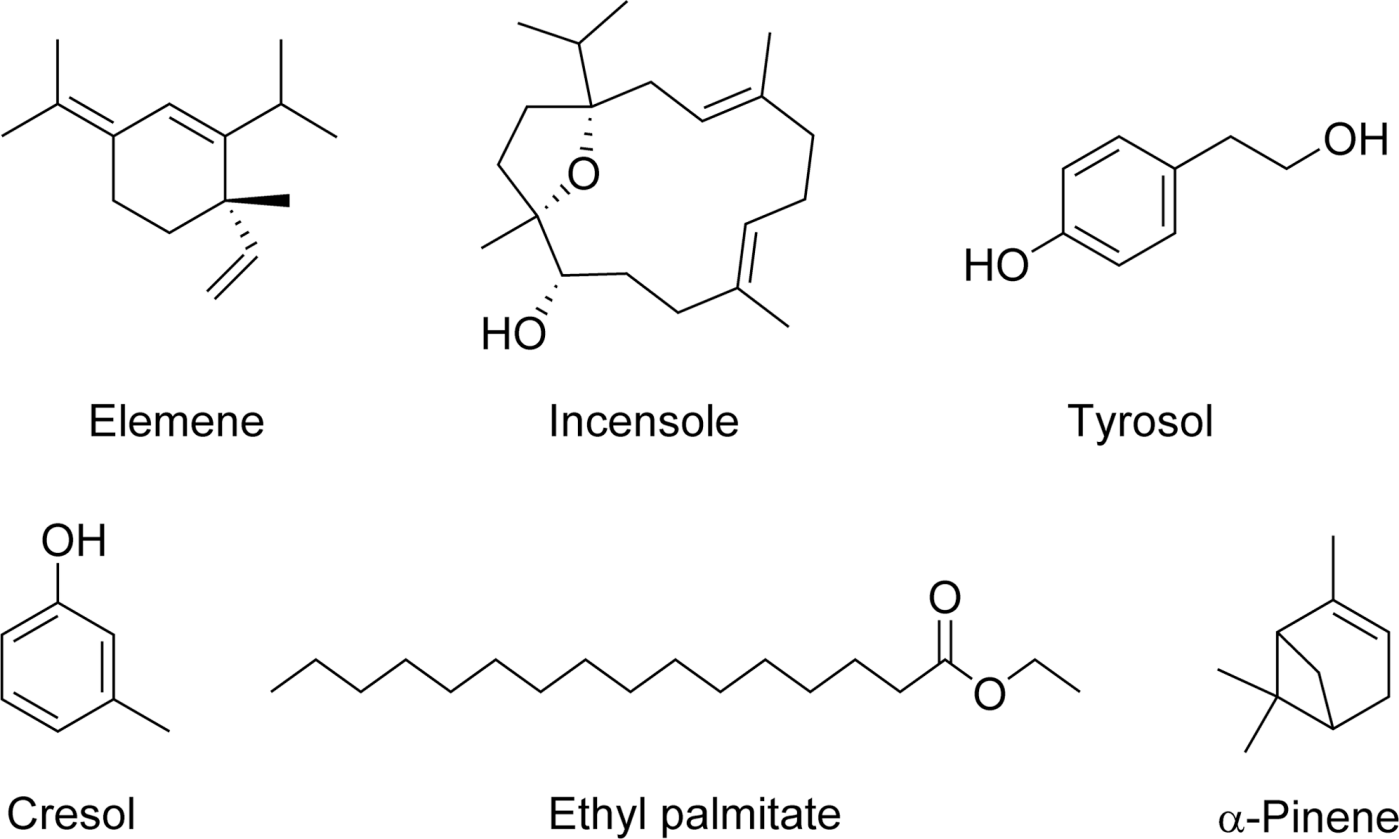
Chemical structures of selected compounds found from gins with (+) APPI.

Since ESI does not efficiently ionize most nonpolar compounds, APPI was used as a complementary ionization method to cover a wider range of compounds. Positive-ion APPI FT-ICR mass spectra for three gin samples G1, G2, and G10 are shown in Figure 3 (for the APPI spectra of the rest of the samples, see Figures S3 and S4). Characteristic peaks in many samples were observed at *m*/*z* 136.124652 and 204.187244, representing various monoterpenes and sesquiterpenes, respectively. The most abundant monoterpenes in gins are limonene, pinene, and myrcene, whereas caryophyllene, cadinene, and elemene are the main sesquiterpenes reported previously.^3^ Further separation of these compounds requires GC–MS. Either radical cations M^+•^ or protonated molecules [M + H]^+^ (or both) are observed using (+) APPI, depending on the proton and electron affinities. Only the most abundant ion type for each detected molecule is reported here. Figure 4 depicts the chemical structures of six selected compounds found from gins with positive-ion APPI.

When compared with (−) ESI, a slightly higher average number of unique spectral features (∼1600 without G10) could be detected with (+) APPI, when combining radical and nonradical ions. Again, the gin sample G10 showed the highest number of spectral features (>3100) due to its high content of dissolved phenolic compounds (Figure S5). Therefore, it is evident that barrel maturation considerably increases the chemical diversity of gin.

The Supporting Information data (Table S2) presents 47 tentatively identified compounds in gin samples by (+) APPI FT-ICR MS, including mono-, sesqui-, and diterpenes, phenolic compounds, alcohols, ketones, aldehydes, and esters. Especially, different fatty acid esters and terpenes were the most abundant compounds detected with (+) APPI. The APPI-based chemical fingerprints were more unique among different gin samples as compared to ESI, although most of the identified compounds could be detected in every sample. Only six of these 47 compounds have been reported earlier. Many compounds detected with APPI also contribute strongly to the sensory profiles of gins.^3^ However, some compounds were difficult to annotate solely based on the molecular formulae. Additional tandem mass spectrometry experiments, for example, could be used for more confident assignments.

### 3.3 Data Visualization: van Krevelen Diagrams

**Figure 5:**
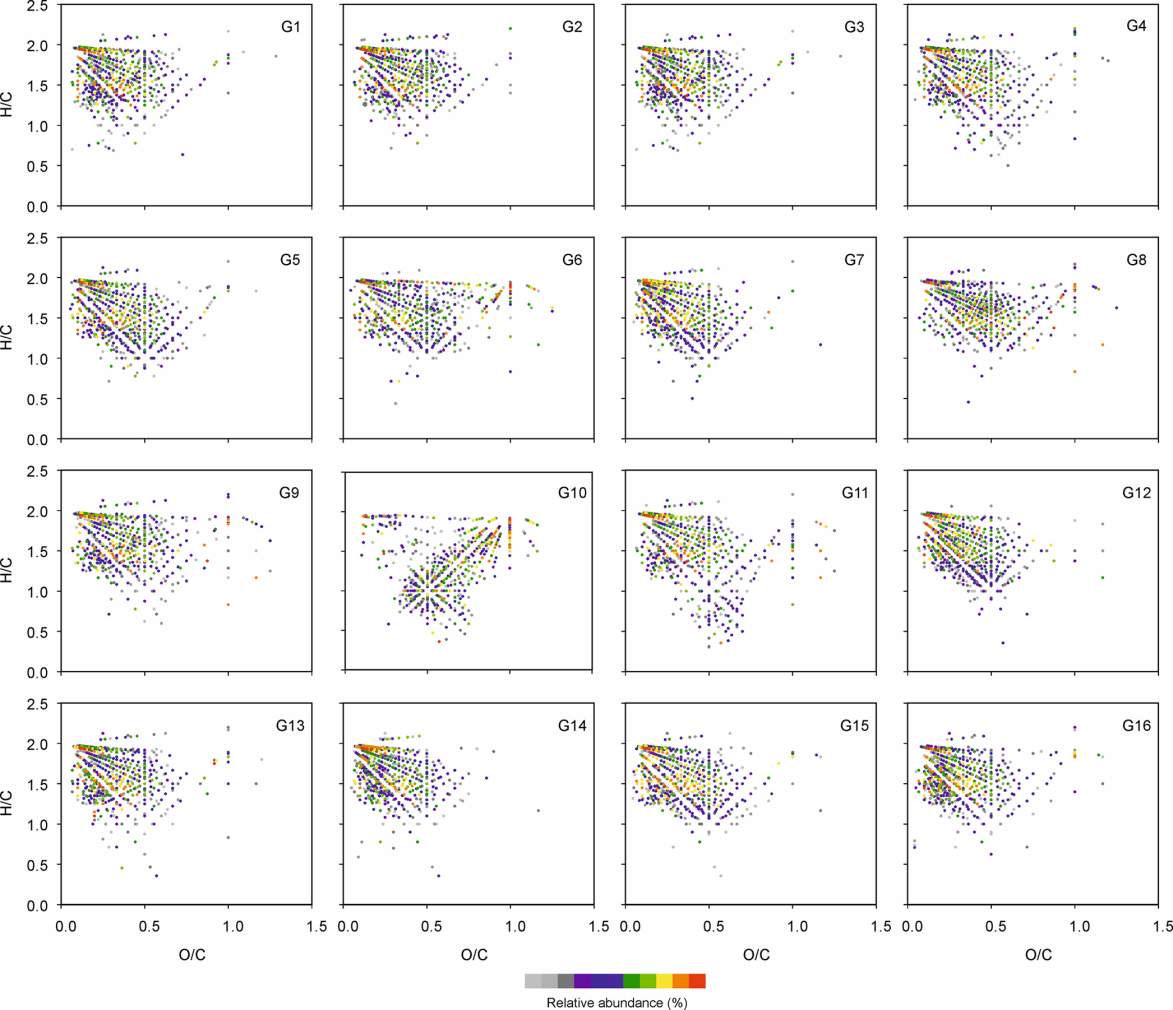
Color-coded van Krevelen diagrams of the compounds present in 16 gins based on the negative-ion ESI FT-ICR MS data.

A van Krevelen diagram is a plot of the atomic hydrogen-to-carbon (H/C) versus oxygen-to-carbon (O/C) ratio for each detected compound in each sample. It can be used to visualize the composition of a complex organic sample in a chemically relevant manner because different compound classes can be differentiated by their respective H/C and O/C ratios (e.g., lipids, sugars, phenolics, amino acids, and condensed aromatics). However, plain hydrocarbons do not separate well in VK diagram as they line up within the *y*-axis of the diagram (O/C = 0). Therefore, we only visualized the (−) ESI data of the gin samples using VK diagrams (Figure 5). The van Krevelen diagrams reveal several common features in all of the studied gin samples. The species located on the top left corner (H/C ≥ 1.6, O/C ≤ 0.3) correspond to aliphatic compounds, especially organic acids. The species located in the top right corner (H/C ≥ 1.6, O/C ≥ 0.6) are different carbohydrates. The species in the middle of the diagram (H/C ≈ 1–1.6, O/C ≈ 0.2–0.6) are phenolic compounds and their derivatives, especially abundant in G10. There are no marked differences between VK diagrams of the other samples; the samples G6, G8, and G10 show higher abundance for compounds with high O/C ratios, consistent with the higher content of sugars.

### 3.4 Principal Component Analysis

The PCA analysis was performed to evaluate data reproducibility and to analyze sample variance and clustering. For the reproducibility evaluation, three selected gins (G1, G2, G10) were analyzed five times (technical replicates), and the two-dimensional PCA analysis was conducted with the identified compounds. Figure S6 shows the PCA scores plot for G1, G2, and G10. It can be seen that the five replicate measurements (A–E) are tightly clustered and that all three gin samples can be well separated by PCA. In the PCA analysis of all 16 gins, three cluster groups were formed (Figure S7). The first group contains gins G1, G2, G3, G4, G8, G9, G11, and G16. The rest of the gins are clustered toward the lower part of the plot, except G10 (barrel-aged Koskue), which is clearly separated from all of the other gins. The other gins were clustered mainly according to the amount of terpenoids, acids, and sugars with moderate statistical significance. For example, gins G2, G8, G9, G11, and G16 had clearly a lower content of terpenoids, and they belonged to the first cluster.

## 4 Conclusions

Direct-infusion ultrahigh-resolution FT-ICR mass spectrometry was successfully applied for the first time to analyze gin, one of the largest-selling distilled spirits worldwide. Gin derives its unique taste from natural botanicals, which contribute to its distinct flavor among different alcoholic beverages. The chemical fingerprints of 16 commercial gins were obtained using direct-infusion ESI/APPI FT-ICR mass spectrometry, allowing identification of 135 volatile and nonvolatile compounds, out of which 122 have not been reported earlier. Especially, the nonvolatile constituents in gins have been very poorly characterized to date. The main compounds identified in this work were mono-, sesqui-, and diterpenes, terpenoids, phenolic compounds, organic acids, esters, and ketones/aldehydes. Some compounds could be attributed to specific ingredients or production methods. For example, the barrel-aged gin was distinguished by the high content of oak wood-derived phenolics. While direct-infusion FT-ICR MS provides a rapid chemical fingerprinting of distilled spirits to identify the main flavor components and to verify product quality and authenticity, several structural isomers present in the samples cannot be differentiated by accurate mass only, and thus additional hyphenated techniques, e.g., GC–MS or LC–MS, or the use of tandem mass spectrometry are needed.

## Supporting Information Available

Supporting mass spectra, PCA plots, and compound identification tables (PDF)

## Notes

Any brand names appearing in this article are for the product identification purpose only.
