# Supplementary material for: Analysis of Volatile and Nonvolatile Constituents in Gin by Direct-Infusion Ultrahigh-Resolution ESI/APPI FT-ICR Mass Spectrometry
Source: J Agric Food Chem. 2023 Apr 27;71(18):7082–9. doi: 10.1021/acs.jafc.3c00707 (PMC10176568; doi:10.1021/acs.jafc.3c00707)
Supplement: Supplementary file 1 — jf3c00707_si_001.pdf [file jf3c00707_si_001.pdf]

Supplementary Information

Analysis of Volatile and Nonvolatile Constituents in Gin by Direct-Infusion Ultrahigh-Resolution ESI/APPI FT-ICR Mass Spectrometry

*Yanning Dou, Marko Mäkinen & Janne Jänis**

Department of Chemistry, University of Eastern Finland, P.O. Box 111, FI-80101 Joensuu, Finland

**Corresponding author E-mail: janne.janis@uef.fi*

Contents:

Figure S1. (–) ESI-FT-ICR mass spectra of the gin samples G3–G9. 2

Figure S2. (–) ESI-FT-ICR mass spectra of the gin samples G11–G16. 3

Figure S3. (+) APPI-FT-ICR mass spectra of the gin samples G3–G9. 4

Figure S4. (+) APPI-FT-ICR mass spectra of the gin samples G11–G16. 5

Figure S5. Number of assigned peaks in the mass spectra of gin samples G11–G16....................6

Figure S6. PCA scores plot of the three gin samples G1, G2, and G10 with technical replicates A–E....................7

Figure S7. PCA scores plot of the gin samples G1–G16 analyzed in this study....................8

Table S1. Compounds tentatively identified in the gin samples G1–G16 by (–) ESI FT-ICR MS. 9

Table S2. Compounds tentatively identified in the gin samples G1–G16 by (+) APPI FT-ICR MS. 12

Table S3. Calibration mass list for (–)ESI 14

Table S4. Calibration mass list for (+)APPI....................15

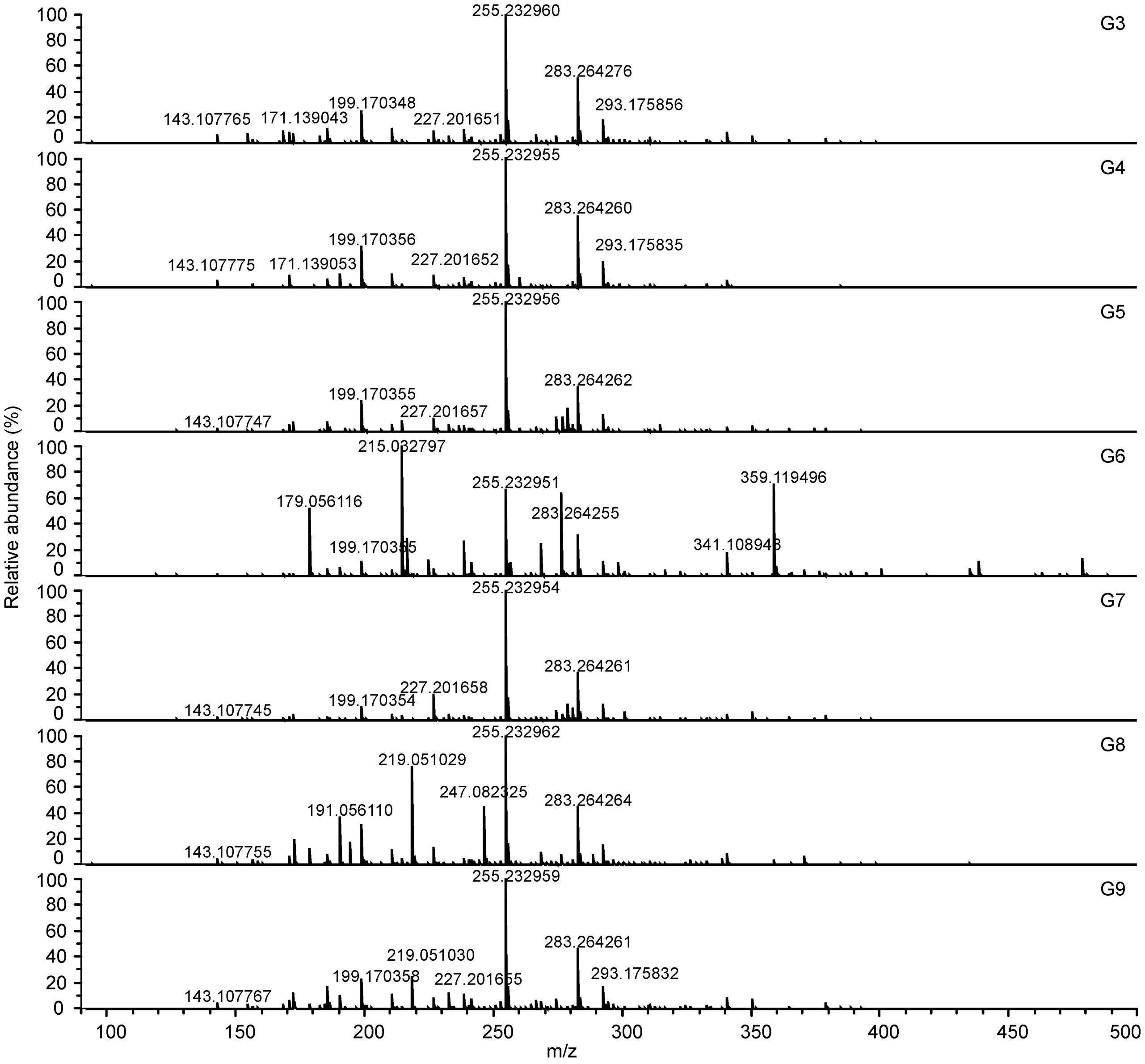

Figure S1. *(–) ESI-FT-ICR mass spectra of the gin samples G3–G9.*

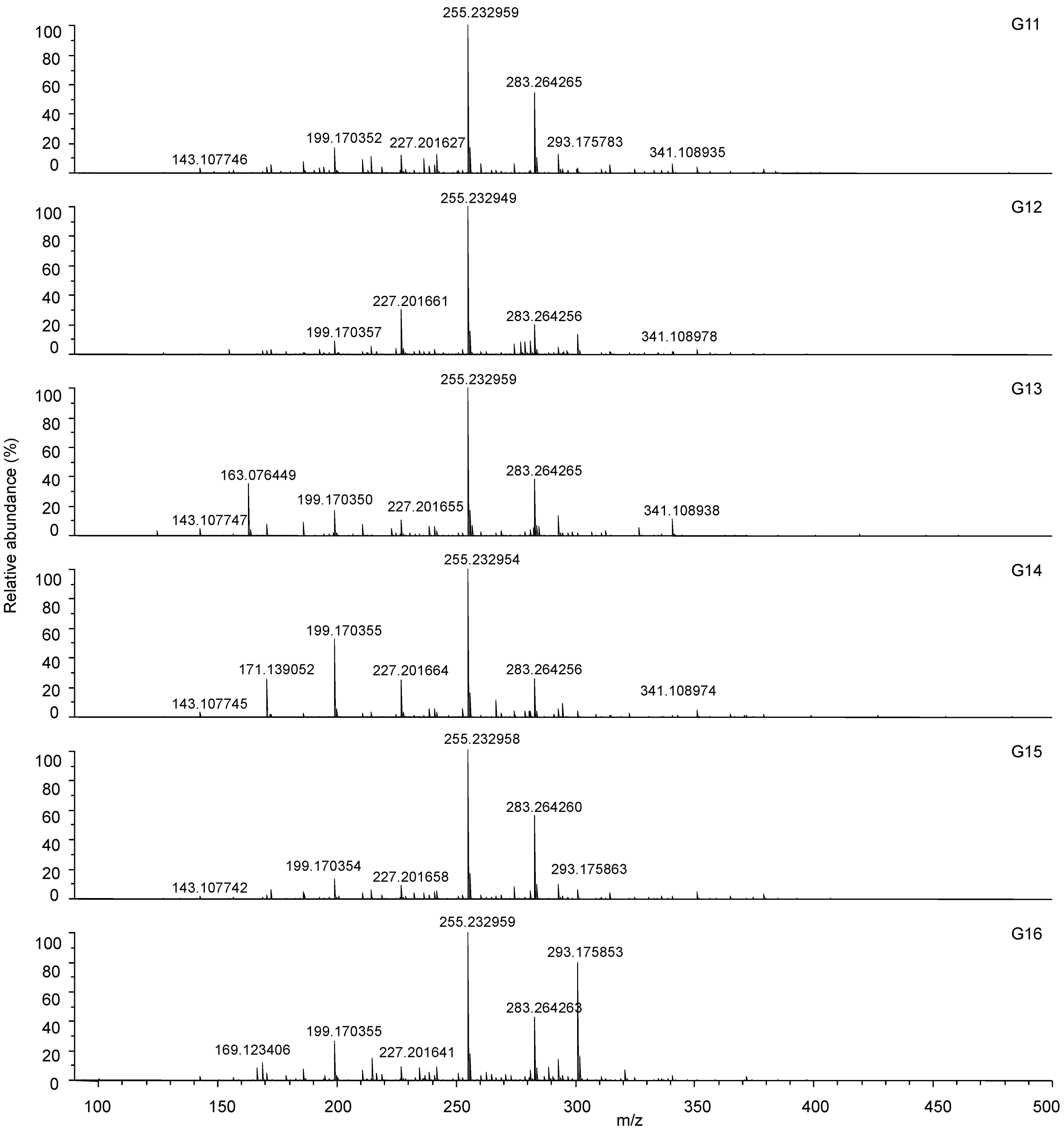

Figure S2. *(–) ESI-FT-ICR mass spectra of the gin samples G11–G16.*

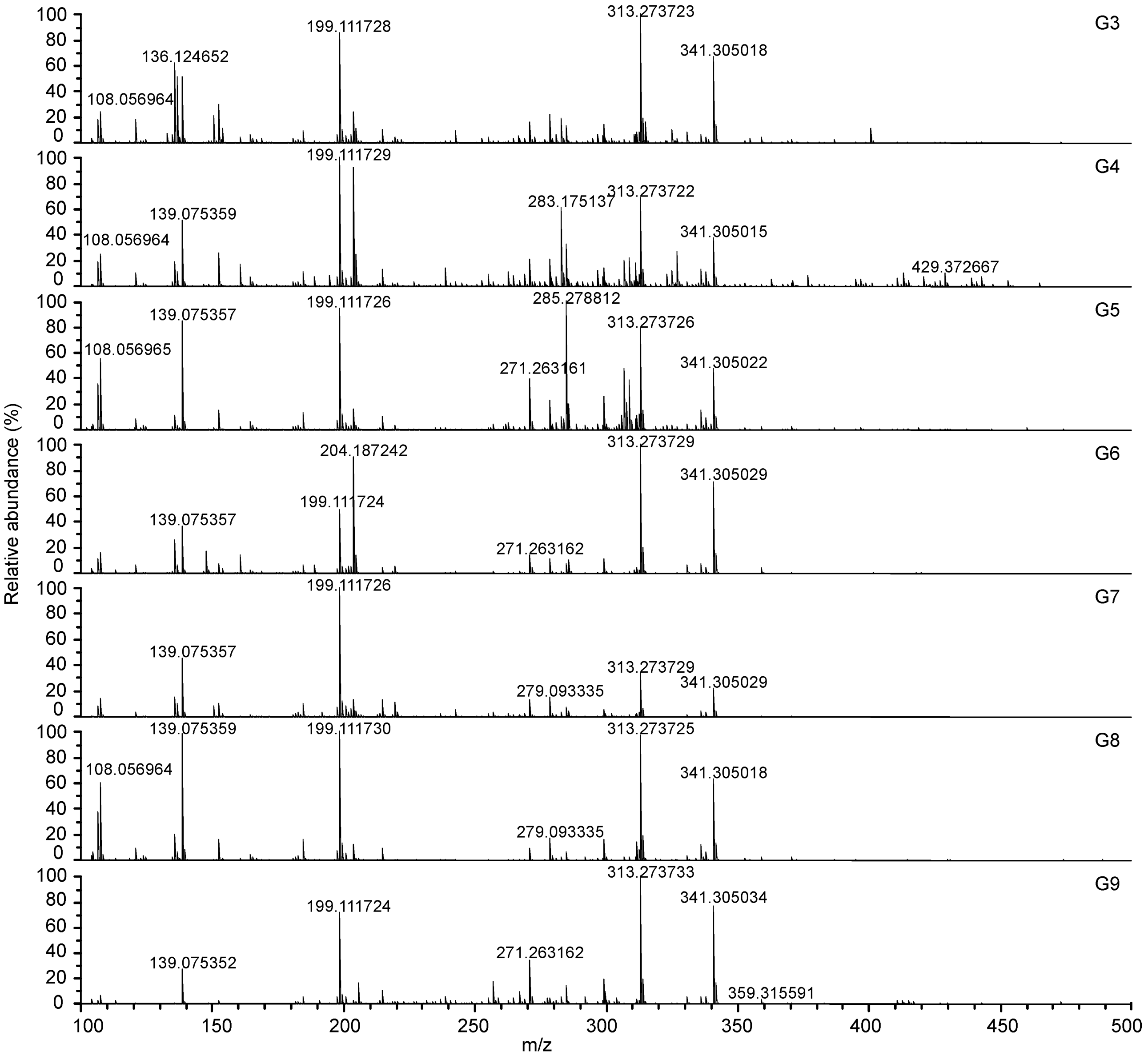

Figure S3. *(+) APPI-FT-ICR mass spectra of the gin samples G3–G9.*

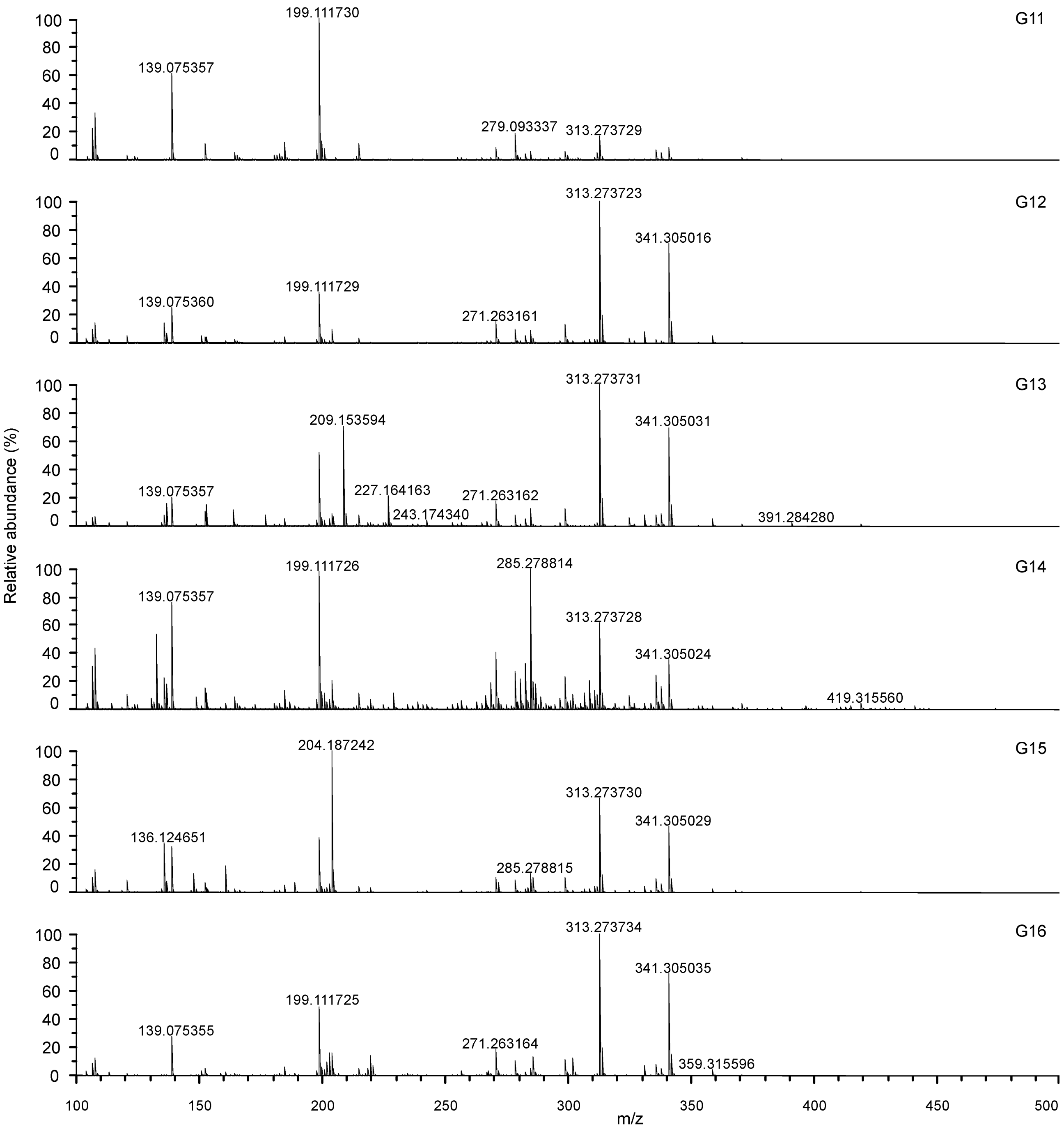

Figure S4. *(+) APPI-FT-ICR mass spectra of the gin samples G11–G16.*

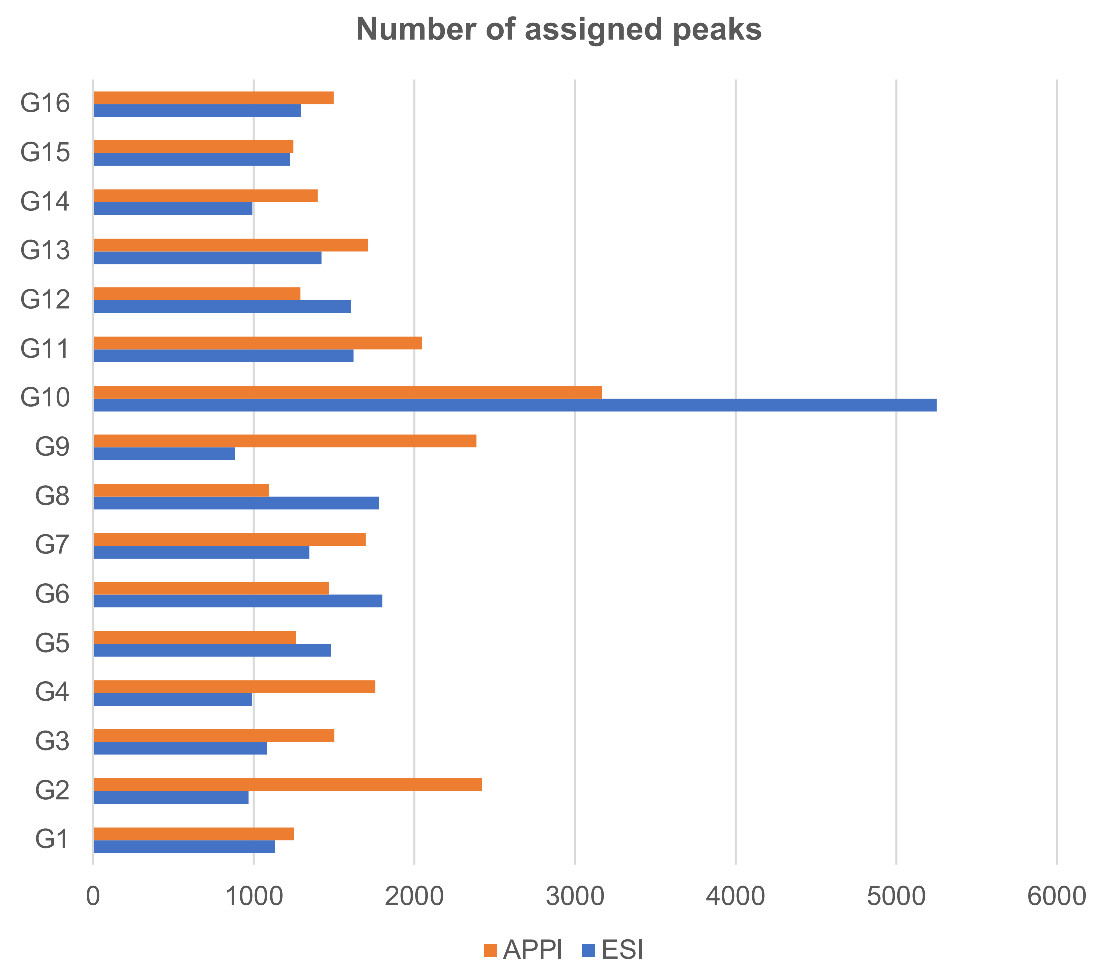

Figure S5. *Number of assigned peaks in the mass spectra of gin samples G11–G16.*

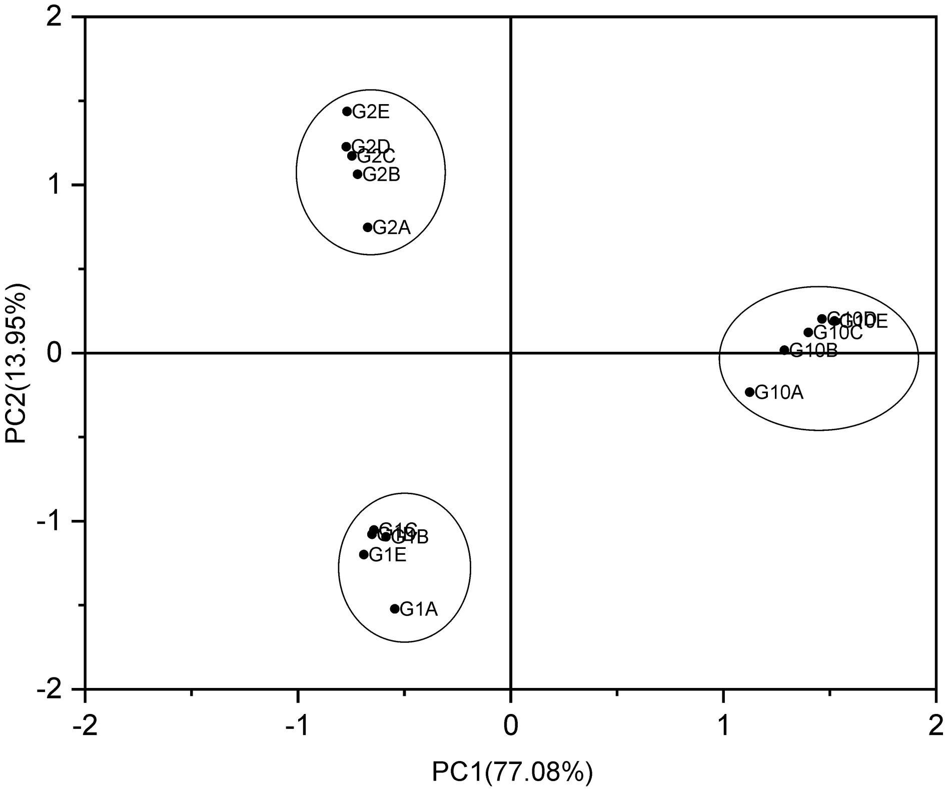

Figure S6. *PCA scores plot of the three gin samples G1, G2, and G10 with technical replicates A–E.*

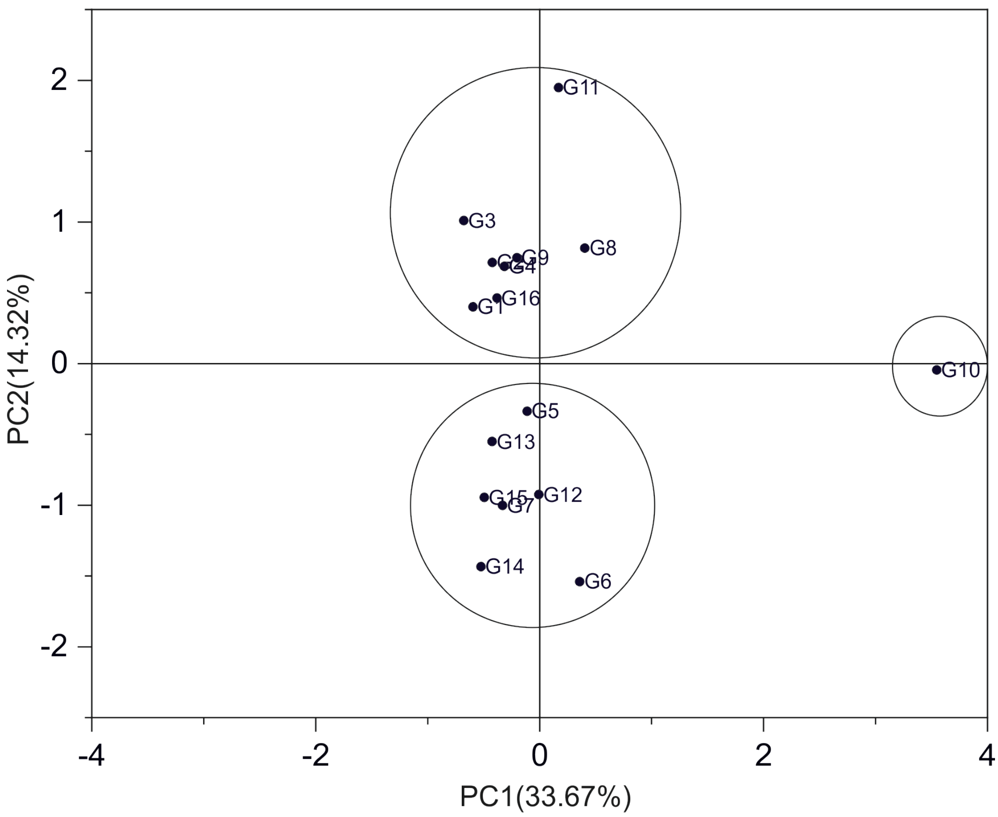

Figure S7. *PCA scores plot of the gin samples G1–G16 analyzed in this study.*

Table S1. *Compounds tentatively identified in the gin samples G1–G16 by (–) ESI FT-ICR MS.*

Group	Ion formula[1]	m/z_{mono}	DBE[2]	Error (ppm)[3]	Compound[4]	G1	G2	G3	G4	G5	G6	G7	G8	G9	G10	G11	G12	G13	G14	G15	G16
						Relative intensity (%)[5]															
Monoterpenoid	$C_{10}H_{13}O$	149.097217	4	0.030	carvone[a]	0.0224	0.0334	0.2674	0.0000	0.0396	0.0155	0.0347	0.0074	0.0000	0.0000	0.0155	0.0512	0.0270	0.0212	0.0489	0.1361
	$C_{10}H_{15}O$	151.112873	3	0.034	camphor[b]	0.2566	0.0282	0.0460	0.0300	0.0961	0.0598	0.0692	0.0087	0.0000	0.0000	0.0198	0.0213	0.0590	0.0173	0.1530	0.3158
	$C_{10}H_{17}O$	153.128526	2	0.036	linalool[c]	0.2912	0.0564	0.2104	0.1649	0.2142	0.0539	0.4578	0.0000	0.0000	0.0055	0.0920	0.0404	0.4605	0.1501	0.1693	0.0319
Sesquiterpenoid	$C_{14}H_{21}O$	205.159801	4	0.017	methylionone	0.1687	0.1598	0.1685	0.2041	0.1757	0.0412	0.0804	0.0746	0.1182	0.0107	0.1795	0.0446	0.1343	0.1969	0.1672	0.1297
	$C_{15}H_{23}O$	219.175494	4	0.021	spathulenol[b]	0.5947	0.0768	0.1992	0.1282	0.3210	0.4206	0.4710	0.0119	0.2993	0.0066	0.0442	0.3416	0.1558	0.2069	1.3873	1.5881
	$C_{15}H_{25}O$	221.191157	3	0.025	elemol[b]	0.0821	0.0489	0.0958	0.0352	0.0811	0.1220	0.1677	0.0000	0.0000	0.0069	0.0165	0.1480	0.1584	0.0564	0.3582	0.1911
	$C_{15}H_{23}O_3$	251.165253	4	0.019	capnellane sesquiterpene	1.2724	1.2630	1.1625	1.5583	1.0202	0.4351	0.5659	0.4009	1.1036	0.0532	0.8891	0.7057	0.8176	0.3985	0.9152	1.7929
	$C_{16}H_{25}O_3$	265.180944	4	0.027	juvabione	0.1704	0.1329	0.1617	0.1085	0.1257	0.1936	0.2442	0.0000	0.0369	0.0578	0.0375	0.2681	0.2419	0.0980	0.4425	0.4114
Diterpenoid	$C_{20}H_{35}O_2$	307.264253	3	0.036	sclareol	0.1339	0.0249	0.1123	0.0408	0.0494	0.0279	0.3183	0.0000	0.0000	0.0297	0.0130	0.0234	0.2734	0.1226	0.1010	0.0204
Phenolics	$C_7H_5O_3$	137.024438	5	0.090	salicylic acid	0.0174	0.0000	0.0251	0.0246	0.0137	0.0056	0.0605	0.0118	0.0194	0.0474	0.0349	0.0832	0.0110	0.0050	0.0095	0.0155
	$C_8H_7O_3$	151.040065	5	0.077	vanillin[c]	0.0155	0.0954	0.0334	0.1638	0.0394	0.0049	0.0224	0.0303	0.0000	0.2040	0.0456	0.0527	0.0176	0.0056	0.0103	0.0260
	$C_{10}H_{11}O_2$	163.076518	5	0.062	eugenol[d]	0.0306	0.0308	0.0140	0.0537	0.0104	0.0046	0.0252	0.0112	0.0000	0.0000	0.0198	0.0224	12.4628	0.0096	0.0186	0.0485
	$C_8H_7O_4$	167.035003	5	0.039	vanillic acid	0.0240	0.0442	0.0257	0.1356	0.1024	0.0160	0.0175	0.0000	0.0478	0.2212	0.1049	0.0295	0.0255	0.0059	0.0217	0.0416
	$C_7H_5O_5$	169.014233	5	0.064	gallic acid	0.0000	0.0000	0.0000	0.0000	0.0066	0.0050	0.0275	0.0124	0.0000	0.5510	0.3355	0.0870	0.0304	0.0047	0.0120	0.0573
	$C_{10}H_9O_3$	177.055716	6	0.027	coniferyl aldehyde	0.0195	0.0357	0.0310	0.0969	0.1469	0.0104	0.0198	0.0222	0.0324	0.6188	0.0976	0.0367	0.0270	0.0168	0.0163	0.0471
	$C_{10}H_{11}O_3$	179.071437	5	0.019	coniferyl alcohol	0.1793	0.3397	0.2251	0.2313	0.1343	0.0428	0.0684	0.0996	0.1561	0.0261	0.1332	0.0631	0.1125	0.0466	0.0913	0.2048
	$C_9H_9O_4$	181.050639	5	0.031	syringaldehyde	0.0224	0.0527	0.0395	0.0729	0.2372	0.0241	0.0234	0.0264	0.0312	0.9445	0.1468	0.0562	0.0302	0.0124	0.0267	0.0416
	$C_{10}H_9O_4$	193.050641	6	0.021	ferulic acid	0.0564	0.0583	0.0708	0.0854	0.3195	0.0226	0.0588	0.0916	0.0614	0.8321	0.1485	0.0697	0.0446	0.0231	0.0445	0.0861
	$C_{11}H_{13}O_3$	193.087102	5	0.041	methoxyeugenol	0.0789	0.0632	0.0957	0.0764	0.0523	0.0217	0.0437	0.0489	0.0718	0.0117	0.0725	0.0510	0.0583	0.0283	0.0527	0.1005
	$C_9H_9O_5$	197.045572	5	0.036	ethyl gallate	0.0243	0.0276	0.0304	0.0519	0.1017	0.0211	0.0268	0.0553	0.0474	1.9271	0.3281	0.0739	0.0488	0.0125	0.0393	0.0499
	$C_{11}H_{11}O_4$	207.066280	6	0.030	sinapaldehyde	0.0338	0.0458	0.0308	0.0371	0.0457	0.0146	0.0324	0.0338	0.0334	2.1719	0.2233	0.0637	0.0374	0.0143	0.0328	0.0771
	$C_{17}H_{25}O_4$	293.175837	5	0.060	gingerol	6.9835	5.9935	7.2454	7.4216	5.5056	2.6574	5.1228	3.3641	6.1592	9.2479	5.2847	2.2339	5.0086	2.2773	4.3000	5.0691
	$C_{14}H_5O_8$	300.998985	12	0.040	ellagic acid	0.0000	0.0000	0.0000	0.0000	0.0000	0.0000	0.0000	0.0000	0.0000	6.0029	0.0126	0.0017	0.0000	0.0028	0.0000	0.0201
Lactone	$C_{10}H_{17}O_2$	169.123459	2	0.021	decalactone	0.4862	0.0000	1.4114	0.1493	0.1730	0.3579	0.4745	0.1321	0.0618	0.0297	0.0777	0.0000	0.4706	0.1758	0.8889	4.2102
	$C_6H_9O_6$	177.040455	2	0.028	gluconolactone	0.0139	0.0412	0.0273	0.0283	0.0171	0.0209	0.0077	0.0393	0.0403	1.3993	0.6613	0.0631	0.0651	0.0110	0.0235	0.0000
	$C_{12}H_{17}O_2$	193.123483	4	0.015	sedanolide	0.1309	0.1651	0.1220	0.1659	0.0730	0.0239	0.0495	0.0663	0.0509	0.0000	0.1067	0.0604	0.1918	0.0245	0.0674	0.1749
Ether	$C_{11}H_{13}O_2$	177.092116	5	0.054	methyl eugenol	0.0261	0.0417	0.0591	0.0193	0.0138	0.0057	0.0098	0.0176	0.0208	0.0000	0.2923	0.0169	0.0169	0.0256	0.0116	0.0374
	$C_{12}H_{15}O_3$	207.102775	5	0.049	asarone	0.0505	0.0277	0.0494	0.0345	0.0454	0.0165	0.0361	0.0291	0.0434	0.0066	0.0407	0.0652	0.7150	0.0214	0.0416	0.0797
Acid	$C_7H_5O_2$	121.029489	5	0.085	benzoic acid	0.0532	0.0431	0.0747	0.0466	0.0295	0.0355	0.0346	0.0362	0.0645	0.0079	0.0576	0.0272	0.0271	0.0056	0.0193	0.0435
	$C_4H_7O_5$	135.029909	1	0.021	threonic acid	0.0000	0.0242	0.0000	0.0000	0.0067	0.0055	0.0000	0.0088	0.0183	0.0393	0.0541	0.0000	0.0090	0.0000	0.0000	0.0000
	$C_8H_7O_2$	135.045177	5	0.049	phenylacetic acid	0.0000	0.0226	0.0167	0.0000	0.0000	0.0000	0.0054	0.0092	0.0000	0.0053	0.0092	0.0130	0.0000	0.0000	0.0000	0.0185
	$C_8H_{15}O_2$	143.107741	1	0.059	caprylic acid	2.0936	1.4375	2.5667	2.1201	1.3273	0.5392	1.1923	1.0931	1.8694	0.0829	1.6082	0.4645	1.9298	1.4476	1.1129	1.1136
	$C_9H_7O_2$	147.045190	6	0.037	cinnamic acid	0.0000	0.0238	0.1723	0.0000	0.0000	0.0035	0.0000	0.0000	0.0000	0.0089	0.0112	0.0111	0.0000	0.0900	0.0000	0.0000

Table S1. *Continued…*

	$C_4H_5O_6$	149.009130	2	0.047	tartaric acid	0.0000	0.0000	0.0000	0.0000	0.0000	0.0000	0.0000	0.0000	0.0000	0.0572	0.7497	0.0681	0.0680	0.0102	0.0222	0.0236
	$C_9H_9O_2$	149.060842	5	0.057	phenylpropanoic acid	0.0000	0.0000	0.0332	0.0000	0.0073	0.0000	0.0000	0.0000	0.0000	0.0036	0.0102	0.0058	0.0000	0.0089	0.0000	0.0120
	$C_9H_{17}O_2$	157.123403	1	0.040	nonanoic acid	1.0363	1.0014	1.1022	1.0758	0.5472	0.2094	0.4237	0.5021	0.9136	0.0231	1.0945	0.3110	0.5713	0.2277	0.6940	0.7950
	$C_6H_7O_5$	159.029942	3	0.041	oxoadipic acid	0.0199	0.0000	0.0152	0.0285	0.0094	0.0061	0.0075	0.0000	0.0254	0.0466	0.0285	0.0132	0.0152	0.0000	0.0071	0.0175
	$C_7H_{11}O_4$	159.066328	2	0.031	pimelic acid	0.3154	0.3234	0.3926	0.3391	0.1609	0.0721	0.1444	0.6515	0.4082	0.0578	0.3477	0.1075	0.1693	0.0384	0.1372	0.2785
	$C_8H_{15}O_3$	159.102704	1	0.029	hydroxyoctanoic acid	0.2417	0.2044	0.2889	0.2470	0.1226	0.0362	0.1174	0.1169	0.2585	0.0072	0.2390	0.0597	0.1254	0.0304	0.0832	0.1644
	$C_8H_5O_4$	165.019363	6	0.060	terephthalic acid	0.0204	0.0204	0.0230	0.0365	0.0181	0.0087	0.0140	0.0360	0.0276	0.0294	0.0820	0.0124	0.0333	0.0060	0.0000	0.0718
	$C_5H_9O_6$	165.040522	1	0.035	xylonic acid	0.0000	0.0000	0.0000	0.0428	0.0189	0.0000	0.0157	0.0399	0.0624	0.1278	0.3343	0.0211	0.0279	0.0000	0.0120	0.0198
	$C_{10}H_{19}O_2$	171.139100	1	0.020	capric acid	2.6613	3.2657	3.6304	3.5751	2.6092	0.4607	1.3846	1.5517	2.5671	0.1055	1.9004	1.3228	2.9993	9.7242	1.4130	1.9772
	$C_7H_9O_5$	173.045540	3	0.022	shikimic acid	0.0897	0.0937	0.0000	0.1127	0.0548	0.0000	0.0423	0.6316	0.1276	0.1056	0.1148	0.0641	0.0672	0.0147	0.0583	0.1390
	$C_7H_{11}O_5$	175.061242	2	0.015	isopropylmalic acid	0.0878	0.1137	0.0993	0.0959	0.0756	0.0229	0.0698	0.2043	0.1178	0.4094	0.1907	0.1605	0.0967	0.0206	0.0832	0.1548
	$C_9H_{15}O_4$	187.097658	2	0.019	azelaic acid	0.5797	0.6205	0.5795	0.5011	0.3584	0.1818	0.3646	0.6846	0.6965	0.1132	0.7708	0.3648	0.4675	0.1167	0.2480	0.5322
	$C_8H_{13}O_5$	189.076895	2	0.021	butanedioic acid	0.1115	0.1255	0.1192	0.0000	0.0938	0.0399	0.0990	0.1909	0.1382	0.1105	0.1427	0.1136	0.0780	0.0194	0.0683	0.1509
	$C_7H_{11}O_6$	191.056114	2	0.021	quinic acid	0.0361	0.0786	0.0599	3.9598	0.3134	1.5507	0.5975	8.0374	3.9146	0.4144	0.4911	0.4780	0.2585	0.0333	0.1318	0.1663
	$C_6H_{11}O_7$	195.051055	1	0.037	gluconic acid	0.0000	0.0000	0.0000	0.0389	0.0275	0.0206	0.0000	0.0380	0.0611	0.5751	0.6582	0.0541	0.0000	0.0101	0.0215	0.0425
	$C_{12}H_{23}O_2$	199.170382	1	0.021	dodecanoic acid	8.3676	13.4009	9.9804	11.4264	9.7576	2.5284	4.6798	6.6964	8.0985	0.4609	7.1227	4.1537	6.1526	19.2989	5.8031	9.3817
	$C_8H_{13}O_6$	205.071846	2	0.046	diethyl tartrate	0.0599	0.1607	0.1169	0.0615	0.0399	0.0211	0.1188	0.1059	0.0634	0.2368	0.0817	0.5875	0.1271	0.0150	0.0390	0.1217
	$C_{13}H_{25}O_2$	213.186037	1	0.011	tridecanoic acid	0.3737	0.6537	0.4709	0.4482	0.3494	0.1229	0.3782	0.3030	0.3790	0.0449	1.0100	0.6318	0.3677	0.3474	0.3588	0.4171
	$C_8H_{11}O_7$	219.051029	3	0.026	dimethylcitric acid	0.0162	0.0000	0.0248	0.0274	0.0227	0.2098	0.0957	16.4768	8.6391	2.6922	1.9114	0.2682	0.1670	0.0000	0.0678	0.0000
	$C_7H_{13}O_8$	225.061593	1	0.039	glucoheptonic acid	0.0151	0.0000	0.0246	0.0166	0.0203	2.7790	0.0162	0.3756	0.2158	1.2236	0.0395	0.0124	0.0153	0.0000	0.0538	0.2568
	$C_{14}H_{25}O_2$	225.186014	2	0.018	myristoleic acid	0.0929	0.2503	0.1153	0.0920	0.1380	0.0688	1.0234	0.1168	0.0624	0.1483	0.0820	2.0166	0.1293	0.9288	0.2293	0.3221
	$C_{14}H_{27}O_2$	227.201707	1	0.032	myristic acid	3.6386	4.9770	3.7580	3.5795	4.5559	1.3269	8.6260	2.9379	3.1543	1.0032	4.9540	13.4148	3.8722	9.4309	3.9946	3.4816
	$C_{10}H_{13}O_6$	229.071818	4	0.016	dioxodecanedioic acid	0.0000	0.0908	0.0966	0.0995	0.1310	0.0654	0.0727	0.3467	0.0000	0.3763	0.1195	0.1165	0.0677	0.0252	0.0882	0.1268
	$C_{12}H_{21}O_4$	229.144656	2	0.038	dodecanedioic acid	0.8772	0.6593	1.3067	0.4488	0.4084	0.1552	0.3363	0.5878	0.4762	0.1383	0.5008	0.6700	0.4637	0.1584	0.2754	0.6156
	$C_{11}H_{13}O_6$	241.071674	5	0.010	elenolic acid	0.0737	0.0837	0.0000	0.0000	0.1511	0.0696	0.0786	0.1786	0.0000	1.8780	0.1383	0.1237	0.0606	0.0219	0.0827	0.0802
	$C_{10}H_{13}O_7$	245.066567	4	0.025	*glutaric acid dimer*	0.0409	0.0463	0.0513	0.0416	0.0638	0.0392	0.0000	0.1823	0.0699	0.6229	0.0682	0.0585	0.0303	0.0124	0.0461	0.0000
	$C_{10}H_{15}O_7$	247.082325	3	0.061	butanetricarboxylic acid	0.0565	0.0728	0.0493	0.0423	0.0521	0.2576	0.0352	9.6945	0.3713	3.4409	0.1479	0.0709	0.0432	0.0130	0.0482	0.0000
	$C_{16}H_{29}O_2$	253.217310	2	0.024	palmitoleic acid	0.7461	0.8173	0.7218	0.6088	1.5112	0.2392	1.2732	0.3219	0.4730	0.6152	0.3228	1.8081	0.5184	2.1065	1.4378	0.5828
	$C_{16}H_{31}O_2$	255.232955	1	0.021	palmitic acid	42.2413	39.8129	37.5983	35.2894	40.1047	14.3809	41.6675	22.0468	34.1345	13.1642	39.2370	44.2593	35.0840	36.2857	42.8074	34.6664
	$C_9H_{15}O_9$	267.072126	2	0.057	sialosonic acid	0.0000	0.0000	0.0000	0.0352	0.0219	0.0654	0.0000	0.0000	0.0435	4.0924	0.0481	0.0086	0.0087	0.0000	0.0000	0.0203
	$C_{12}H_{13}O_7$	269.066596	6	0.048	phenyl glucuronide	0.0234	0.0000	0.0000	0.0320	0.0579	0.0360	0.0000	0.0408	0.0253	1.1015	0.0500	0.0618	0.0189	0.0071	0.0400	0.0239
	$C_{17}H_{33}O_2$	269.248563	1	0.020	heptadecanoic acid	0.8016	0.7167	0.6724	0.7201	0.5804	0.3145	0.7453	0.4386	0.6353	0.3891	0.8497	0.6831	1.3750	1.0996	1.2640	0.7471
	$C_{18}H_{29}O_2$	277.217323	4	0.053	linolenic acid	0.0957	0.2985	0.2714	0.1215	0.8131	0.0534	2.1640	0.1624	0.0913	0.3637	0.0600	3.8967	0.4310	0.2207	0.1242	0.3898
	$C_{18}H_{31}O_2$	279.232961	3	0.033	linoleic acid	0.4503	1.0046	0.7190	0.6115	7.7504	0.2953	5.2160	0.5810	0.3905	2.6823	0.2844	3.8167	1.0402	1.6249	0.7297	1.1290
	$C_{18}H_{33}O_3$	297.243514	2	0.049	ricinoleic acid	0.2443	0.2732	0.2292	0.3029	0.3491	0.1164	0.2259	0.3491	0.3743	0.9665	0.2682	0.4829	0.2519	0.1969	0.3057	0.3449
	$C_{18}H_{33}O_2$	281.248596	2	0.022	oleic acid	1.8536	2.0194	1.9023	1.7716	2.3652	0.8410	4.0572	0.8575	1.1142	2.9663	0.9442	4.2372	1.5176	1.7935	2.6135	2.7031
	$C_{18}H_{35}O_2$	283.264258	1	0.039	stearic acid	20.4788	17.6074	19.1511	19.9383	14.3119	6.9418	15.0665	9.6431	15.9472	3.8024	21.9499	8.9946	13.5603	9.7637	23.8285	14.9793

***Table S1.** Continued…*

	$C_{20}H_{39}O_2$	311.295552	1	0.036	arachidic acid	0.2343	0.1831	0.2363	0.2274	0.1788	0.0991	0.2358	0.1300	0.1665	0.1320	0.2286	0.1836	0.4794	0.3499	0.4085	0.2314
	$C_{12}H_{17}O_{10}$	321.08271	4	0.019	peroxydiadipic acid	0.0000	0.0000	0.0000	0.0000	0.0210	0.0096	0.0000	0.0111	0.0000	4.9662	0.1105	0.0199	0.0000	0.0000	0.0000	0.0000
	$C_{24}H_{47}O_2$	367.358160	1	0.051	lignoceric acid	0.0972	0.0635	0.0896	0.0933	0.0564	0.0435	0.0934	0.0565	0.0806	0.1455	0.0757	0.0509	0.4052	0.3887	0.2819	0.1322
Carbohydrate	$C_4H_7O_4$	119.034982	1	0.039	erythrose	0.0115	0.0297	0.0227	0.0000	0.0063	0.0100	0.0000	0.1521	0.0226	0.0066	0.0143	0.0211	0.0000	0.0000	0.0000	0.0000
	$C_5H_7O_4$	131.034990	2	0.063	glutaric acid	0.0165	0.0225	0.0185	0.0179	0.0172	0.0223	0.0116	0.0423	0.0201	0.0597	0.0389	0.0165	0.0214	0.0000	0.0087	0.0317
	$C_6H_9O_4$	145.050620	2	0.056	adipic acid	0.0770	0.0850	0.0805	0.0909	0.0659	0.0457	0.1146	0.2950	0.1036	0.3559	0.1498	0.2293	0.1298	0.0182	0.0634	0.1035
	$C_5H_9O_5$	149.045574	1	0.062	ribose, xylose	0.0000	0.0238	0.0140	0.0241	0.0147	0.0409	0.0100	0.0241	0.0000	0.3671	0.0412	0.0189	0.0142	0.0000	0.0085	0.0388
	$C_6H_9O_5$	161.045509	2	0.045	levoglucosan	0.0350	0.0397	0.0505	0.0412	0.0454	0.3698	0.0232	0.2678	0.0000	0.3897	0.1223	0.0696	0.0447	0.0044	0.0473	0.1476
	$C_8H_{15}O_4$	175.097604	1	0.017	cladinose	0.1553	0.1551	0.2169	0.1191	0.0730	0.0228	0.2832	0.0646	0.1441	0.0385	0.1150	0.3574	0.0957	0.0232	0.0532	0.0904
	$C_6H_{11}O_6$	179.056090	1	0.025	glucose	0.0601	0.0710	0.1728	0.0000	0.2112	11.3623	0.1103	2.8734	1.4198	0.5090	0.0983	0.0386	0.3133	0.0095	0.3242	1.3433
	$C_6H_{12}O_6[Cl^-]$	*215.032789*	*1*	*0.072*	*glucose*	0.0000	0.0000	0.2041	0.1087	0.4242	21.5535	0.1302	0.6392	0.8490	0.0000	0.0000	0.0438	0.1965	0.0354	0.6403	5.3984
	$C_8H_{13}O_7$	221.066738	2	0.049	ethyl glucuronide	0.0120	0.0000	0.0174	0.0293	0.0423	0.0172	0.0079	0.0341	0.0000	1.3194	0.1054	0.0293	0.0162	0.0000	0.0110	0.0506
	$C_8H_{15}O_8$	239.077299	1	0.061	carbohydrate	0.0298	0.0000	0.0900	0.1074	0.0986	2.6950	0.0000	0.4978	0.4560	5.3911	0.0495	0.0443	0.0677	0.0000	0.0691	0.4896
	$C_5H_{11}O_9$	247.012943	2	0.026	ribose	0.0000	0.0000	0.0000	0.0000	0.0126	0.0058	0.0000	0.0266	0.0000	5.0694	0.0388	0.0187	0.0000	0.0000	0.0000	0.0000
	$C_9H_{17}O_9$	269.087772	1	0.059	carbohydrate	0.0408	0.0529	0.0332	0.0581	0.1062	5.5861	0.0000	2.2196	2.1714	2.0995	0.0100	0.0000	0.0793	0.0000	0.1148	0.6797
	$C_{12}H_{19}O_{11}$	339.093207	3	0.072	carbohydrate	0.0000	0.0000	0.0000	0.0000	0.0119	0.0090	0.0000	0.0260	0.0000	6.9443	0.1354	0.0183	0.0080	0.0000	0.0000	0.0000
	$C_{12}H_{21}O_{11}$	341.108934	2	0.080	maltose	0.1510	0.0428	0.3596	0.0292	0.1076	4.1840	0.0480	0.8143	0.0494	2.5414	0.1070	0.0192	4.1807	0.0085	0.1595	0.1011
	$C_{12}H_{23}O_{12}$	359.119500	1	0.071	*glucose dimer*	0.0000	0.0000	0.0000	0.0000	0.0091	15.1573	0.0063	0.9540	0.0520	0.5113	0.0000	0.0000	0.0000	0.0171	0.0085	0.0456

[1] For $[M – H]^-$ ion.
[2] DBE = double bond equivalent (number of rings + double bonds)
[3] Error (ppm): mean (absolute) value of mass error among 16 samples.
[4] Tentative compound identification (Confidence Level 3; Schrimpe-Rutledge et al. *J. Am. Soc. Mass Spectrom.* 2016, 27: 1897.). If several putative compounds exist, the most likely one(s) based on the previous reports has been proposed.
[5] Color: normalized abundance of each compound from red (highest) to green (lowest).

a: Dussort, P., Deprêtre, N., Bou-Maroun, E., Fant, C., Guichard, E., Brunerie, P., … Le Quéré, J.-L. (2012). An original approach for gas chromatography-olfactometry detection frequency analysis: Application to gin. *Food Research International*, *49*(1), 253–262. https://doi.org/10.1016/j.foodres.2012.07.011

b: Vichi, S., Riu-Aumatell, M., Mora-Pons, M., Buxaderas, S., & López-Tamames, E. (2005). Characterization of Volatiles in Different Dry Gins. *Journal of Agricultural and Food Chemistry*, *53*(26), 10154–10160. https://doi.org/10.1021/jf058121b

c: Buck, N., Goblirsch, T., Beauchamp, J., & Ortner, E. (2020). Key Aroma Compounds in Two Bavarian Gins. *Applied Sciences*, *10*(20), 7269. https://doi.org/10.3390/APP10207269

d: Einfalt, D. (2020). Characterization of volatile compounds in quality-ranked gins. *Mitteilungen Klosterneuburg*, *70*(4), 278–291. https://doi.org/10.1021/jf058121b

Table S2. *Compounds tentatively identified in the gin samples G1–G16 by (+) APPI FT-ICR MS.*

Group	Ion Formula[1]	m/z_{mono}[1]	DBE	Error (ppm)	Compound[2]	G1	G2	G3	G4	G5	G6	G7	G8	G9	G10	G11	G12	G13	G14	G15	G16
						Relative intensity (%)															
Monoterpene	$C_{10}H_{14}$	134.109003	4	0.034	cymene	0.2367	0.0268	0.1997	0.1115	0.1330	0.2354	0.1506	0.1752	0.0250	0.0456	0.0642	0.0979	0.0797	0.1473	0.2563	0.1603
	$C_{10}H_{16}$	136.124652	3	0.010	limonene, pinene, myrcene[b]	8.6680	0.0893	9.8958	3.0433	1.7179	5.2110	5.2786	4.1862	0.4644	0.5892	0.5193	4.2342	2.3465	3.2596	7.6393	0.3074
	$C_{10}H_{12}O$	148.088171	5	0.068	estragole, anethole[c]	1.0874	0.0152	0.1754	0.0440	0.0713	3.5238	0.0628	0.0000	0.0212	0.0158	0.0000	0.0281	0.0370	0.1146	3.0922	0.0461
	$C_{10}H_{15}O$ [H]	151.111746	4	0.014	carvone, thymol[b]	0.4818	0.1042	3.5510	0.2417	0.3019	0.3236	2.9053	0.2831	0.0864	0.4232	0.1899	1.7611	0.4651	0.4478	0.3019	1.0624
	$C_{10}H_{17}O$ [H]	153.127397	3	0.031	camphor, decadienal, fenchone[b]	1.3561	0.2666	4.9219	1.0761	0.4065	0.8537	3.2408	0.9285	0.1564	0.3723	0.4821	1.3747	4.3261	1.7517	0.7882	0.5232
	$C_{10}H_{18}O$	154.135223	2	0.032	menthone, decenal	0.9644	0.0511	1.9481	0.2490	0.2194	0.8762	1.2354	0.4282	0.0262	0.1324	0.1336	0.4938	0.6294	0.4482	0.6629	0.2855
Sesquiterpene	$C_{15}H_{23}$ [H]	203.179419	5	0.039	curcumene	1.0759	0.3449	1.1214	1.2727	0.5660	1.1799	2.3576	0.2525	0.2252	0.2968	0.3705	0.5737	1.5874	1.1584	1.3932	4.2688
	$C_{15}H_{24}$	204.187244	4	0.041	caryophyllene, cadinene, elemene[b]	15.7934	0.5534	3.9032	13.9020	2.4482	17.5191	4.7141	2.6797	1.0011	0.5187	0.2906	2.8904	2.5105	3.0355	21.6337	4.4405
	$C_{15}H_{24}O$	220.182162	4	0.026	caryophyllene oxide	0.4649	0.2930	0.8278	0.3992	0.6227	1.2094	3.9214	0.1992	0.3125	0.4746	0.2882	0.4965	0.8019	1.0663	0.8821	4.0621
	$C_{15}H_{24}O_2$	236.177078	4	0.031	farnesoic acid	0.1012	0.0846	0.0908	0.1323	0.0638	0.1056	0.1569	0.1080	0.0955	0.0847	0.0000	0.0421	0.1404	0.0704	0.1253	0.3528
Diterpene	$C_{20}H_{32}$	272.249858	6	0.011	cembrene, abietadiene	0.2516	0.1121	0.1595	0.5930	0.2330	1.0885	0.6271	0.1223	0.1384	0.1760	0.2704	0.2743	0.1195	0.6429	1.6484	0.3764
	$C_{20}H_{31}O$ [H]	287.236954	5	0.030	totarol[e]	1.3336	0.1962	0.2539	0.4514	0.2720	0.2250	0.3997	0.0860	0.1166	0.1121	0.2378	0.2616	0.2907	0.7321	0.3906	0.7589
	$C_{20}H_{35}O_2$ [H]	307.263167	4	0.012	incensole	0.6746	0.1912	0.5202	3.1445	6.9275	0.2875	0.5727	0.6047	0.1637	0.4439	0.3418	0.6656	0.3283	1.7630	0.6472	0.1266
Alcohol	C_8H_9O [H]	121.064790	5	0.010	vinylphenol	0.3291	0.1294	0.2095	0.2209	0.3025	0.2272	0.2113	0.3494	0.1184	0.0973	0.6327	0.1985	0.2017	0.2923	0.2616	0.1729
	$C_{18}H_{35}O$ [H]	267.268248	2	0.015	linoleyl alcohol, octadecenal	0.3697	2.3181	0.6684	0.8226	0.3776	0.3960	0.8615	0.2706	2.8026	2.7378	0.6161	0.6374	1.1149	0.5506	0.2910	0.8209
Phenolics	C_7H_8O	108.056964	4	0.014	cresol	3.9622	2.2764	3.9255	3.9101	8.0143	3.4011	4.9609	12.1287	1.9007	2.0965	14.4251	4.1896	2.1390	6.2058	3.6740	3.4431
	$C_7H_8O_2$	124.051880	4	0.005	guaiacol	0.3285	0.2187	0.3947	0.4603	0.5260	0.2866	0.4439	0.8051	0.2025	0.1939	1.2307	0.3838	0.3201	0.5788	0.3036	0.3047
	$C_8H_{11}O_2$ [H]	139.075360	4	0.019	tyrosol	7.7115	10.2564	8.3938	7.7677	12.2960	7.3402	14.9767	19.4761	7.4908	7.3790	26.6935	7.1716	5.5500	10.9626	7.0899	7.4261
	$C_9H_{13}O_2$ [H]	153.091009	4	0.018	ethyl guaiacol	1.5902	0.0167	3.4217	4.0656	2.2996	1.5823	3.5155	3.4386	1.0162	0.5196	5.1215	1.4442	2.9366	2.2031	1.6605	1.4962
	$C_{13}H_{13}O_2$ [H]	201.090994	8	0.045	hydroquinone benzyl ether	0.7051	1.9893	1.0287	1.1214	0.9672	0.8378	2.9120	1.2829	1.6826	1.9524	3.5165	0.9689	1.2118	1.0824	0.5962	1.3067
	$C_{14}H_{15}O_2$ [H]	215.106649	8	0.034	lunularin	1.1255	3.5736	1.8270	2.0708	1.5930	1.0717	4.5639	2.0951	3.1368	2.3361	5.3578	1.2675	2.2359	1.7700	0.9769	1.6469
	$C_{20}H_{30}O$	286.229128	6	0.032	ferruginol	0.8932	0.1398	0.3357	0.4672	0.7929	2.1226	1.6155	0.2985	0.0453	0.2505	0.5056	1.2397	0.3255	2.7875	2.4797	3.8390
	$C_{22}H_{39}O$ [H]	319.299553	4	0.021	hexadecylphenol	0.9906	0.1734	0.2934	0.5315	0.4476	0.2641	0.2932	0.4829	0.3064	0.3047	0.6383	0.2001	0.3631	0.6625	0.3722	0.3671
	$C_{29}H_{49}O_2$ [H]	429.372667	6	0.031	tocopherol	0.0400	0.1559	0.3022	1.7118	0.1741	0.0215	0.0955	0.0000	0.1276	1.7122	0.1853	0.0855	0.0384	0.3639	0.0337	0.0617
Ketone	$C_9H_{15}O$ [H]	139.111747	3	0.035	nonadienal	1.3962	0.0929	2.8237	0.2650	0.1781	0.2089	0.9859	0.3182	0.0855	0.1940	0.1757	0.5812	0.3815	0.3469	0.2036	0.1762
	$C_{10}H_{11}O$ [H]	147.080440	6	0.020	phenyl butenone	0.6182	0.0565	0.1374	0.0661	0.0644	0.4943	0.0749	0.0842	0.0576	0.0591	0.1114	0.0472	0.1202	0.1206	0.4356	0.0580
	$C_{10}H_{12}O$	148.088270	5	0.029	phenyl butanone	4.3043	0.0152	0.1754	0.0440	0.0713	3.5238	0.0628	0.0000	0.0212	0.0158	0.0000	0.0281	0.0370	0.1146	3.0922	0.0461
	$C_{11}H_{19}O$ [H]	167.143048	3	0.016	dihydrojasmone	0.3660	0.1823	0.4918	0.3447	0.1619	0.3554	0.3168	0.1466	0.1782	0.1036	0.1791	0.1340	0.3338	0.2040	0.4087	0.2330
	$C_{10}H_{17}O_2$ [H]	169.122310	3	0.037	ascaridole	0.3096	0.2483	0.6867	0.2788	0.2011	0.4467	0.5702	0.2406	0.1555	0.1544	0.2076	0.2266	0.4317	0.2821	0.3164	0.2016
	$C_{16}H_{31}O$ [H]	239.236942	2	0.006	hexadecenal	0.2549	1.6988	0.4016	0.4653	0.4017	0.1917	0.6205	0.2436	1.8166	1.1544	0.3797	0.3929	0.6646	0.4613	0.2147	0.3952
Aldehyde	C_7H_7O [H]	107.049143	5	0.010	benzaldehyde	2.7289	1.0013	2.9541	2.9996	5.2062	2.2858	2.9985	7.5131	1.0113	1.2496	9.9044	3.0119	1.8527	4.4673	2.4850	2.4380
Ester	$C_{16}H_{31}O_2$ [H]	255.231859	2	0.012	ethyl myristoleate	0.2376	1.7273	0.8499	1.5379	0.3772	0.1485	1.0325	0.2268	1.4785	2.0360	0.8240	0.3349	0.6866	0.6717	0.1768	0.3536
	$C_{16}H_{33}O_2$ [H]	257.247509	1	0.009	ethyl myristate	0.2758	5.5721	0.4444	0.6959	0.7826	0.1551	1.3007	0.1724	4.9265	4.5427	0.9124	0.3666	0.8177	1.0220	0.2331	0.4260
	$C_{17}H_{33}O_2$ [H]	269.247514	2	0.013	ethyl pentadecanoate	0.3184	1.2077	0.7625	1.4992	0.4597	0.1866	0.9924	0.2577	1.0871	1.5391	1.1658	0.5688	0.6415	1.6976	0.2224	0.4963

Table S2. *Continued…*

	$C_{17}H_{35}O_2$ [H]	271.263164	1	0.019	isopropyl myristate	2.8479	9.3334	2.8160	3.2922	5.7835	3.0045	4.6185	1.9619	9.5694	6.0375	4.1324	4.3906	5.0277	6.0293	2.4881	5.1375
	$C_{18}H_{35}O_2$ [H]	283.263165	2	0.023	ethyl palmitoleate	0.8917	1.9078	3.1355	8.6448	1.6074	0.4610	1.5430	0.6218	1.6362	3.1154	2.1165	1.5650	1.6718	4.7085	0.7173	0.8458
	$C_{18}H_{37}O_2$ [H]	285.278817	1	0.025	ethyl palmitate	3.7349	4.2348	2.3236	4.9638	14.7000	1.6850	2.7349	1.4441	4.0488	4.3240	2.6692	2.7730	3.4275	14.2157	3.0036	1.5499
	$C_{19}H_{37}O_2$ [H]	297.278818	2	0.028	ethyl margarate	0.4266	0.5429	1.1058	1.8881	0.7214	0.2028	0.4179	0.3877	0.5039	0.7281	0.7910	0.6559	0.9156	1.2469	0.3399	0.2738
	$C_{20}H_{37}O_2$ [H]	309.278817	3	0.022	ethyl linoleate	0.7410	0.1777	0.4151	3.3697	5.5180	0.3261	0.9289	0.6415	0.1656	0.4772	0.3117	0.9364	0.3459	3.0060	0.7011	0.1412
	$C_{20}H_{39}O_2$ [H]	311.294469	2	0.013	vaccenyl acetate	0.4814	0.4180	1.1638	2.7961	1.3432	0.2591	0.7424	0.6964	0.3596	0.8712	0.8453	0.8195	0.5042	1.9438	0.4539	0.2888
	$C_{19}H_{37}O_3$ [H]	313.273731	2	0.013	methyl ricinoleate	15.0127	25.1687	16.1334	10.3800	11.3091	19.1988	10.8162	19.5807	26.8112	25.9553	7.6257	28.6772	26.3724	8.8419	14.3496	26.7712
	$C_{21}H_{41}O_2$ [H]	325.310119	2	0.010	octadecyl acrylate	0.5958	0.3971	1.7913	1.9726	0.7023	0.0612	0.2362	0.3320	0.3805	0.6019	0.4258	1.0815	1.7245	1.4918	0.4837	0.1440
	$C_{19}H_{39}O_4$ [H]	331.284294	1	0.028	glyceryl palmitate	1.1420	1.6931	1.4091	0.6600	0.7368	1.4873	0.8694	0.8945	1.7312	2.6901	0.5243	2.3334	2.3565	0.6983	1.0976	1.9138
	$C_{21}H_{41}O_3$ [H]	341.305030	2	0.063	glycidyl stearate	10.7034	19.4850	10.6422	5.3238	6.9276	13.9385	7.4376	12.5978	20.9449	18.4812	4.2974	19.9558	18.7475	5.1606	10.1919	19.0292
	$C_{21}H_{43}O_4$ [H]	359.315590	1	0.062	glycerol monostearate	0.8004	1.1840	0.8806	0.3702	0.4675	1.0375	0.5410	0.7317	1.2278	2.1296	0.1340	0.0315	1.4662	0.4020	0.7677	1.3039
	$C_{24}H_{39}O_4$ [H]	391.284275	6	0.015	ketolithocholic acid	0.3143	0.0790	0.0869	0.2015	0.1205	0.0466	0.0862	0.1374	0.0908	0.1900	0.1552	0.0750	0.7326	0.0879	0.0566	0.0989
	$C_{26}H_{43}O_4$ [H]	419.315578	6	0.035	ketolithocholic acid, ethyl ester	0.9629	0.0000	0.0000	0.1299	0.3841	0.1052	0.0000	0.0881	0.0500	0.0843	0.0000	0.0321	0.6411	0.6822	0.3599	0.0218

[1] For $M^{+\bullet}$ or $[M + H]^+$ ion; the latter is indicated by [H].
[2] DBE = double bond equivalent (number of rings + double bonds)
[3] Error (ppm) = mean (absolute) value of mass error among 16 samples.
[4] Tentative compound identification (Confidence Level 3; Schrimpe-Rutledge et al. *J. Am. Soc. Mass Spectrom.* 2016, 27: 1897.). If several putative compounds exist, the most likely one(s) based on the previous reports has been proposed.
[5] Color: normalized abundance of each compound from red (highest) to green (lowest).

b: Vichi, S., Riu-Aumatell, M., Mora-Pons, M., Buxaderas, S., & López-Tamames, E. (2005). Characterization of Volatiles in Different Dry Gins. *Journal of Agricultural and Food Chemistry*, *53*(26), 10154–10160. https://doi.org/10.1021/jf058121b
c: Buck, N., Goblirsch, T., Beauchamp, J., & Ortner, E. (2020). Key Aroma Compounds in Two Bavarian Gins. *Applied Sciences*, *10*(20), 7269. https://doi.org/10.3390/APP10207269
e: Vichi, S., Aumatell, M. R., Buxaderas, S., & López-Tamames, E. (2008). Assessment of some diterpenoids in commercial distilled gin. *Analytica Chimica Acta*, *628*(2), 222–229. https://doi.org/10.1016/j.aca.2008.

Table S3. *Calibration mass list for (–)ESI*

Name; m/z value; charge; ion formula

C14H15O4	247.097583	1-	C14H15O4
C15H17O4	261.113233	1-	C15H17O4
C16H19O4	275.128883	1-	C16H19O4
C17H21O4	289.144533	1-	C17H21O4
C18H23O4	303.160183	1-	C18H23O4
C19H25O4	317.175833	1-	C19H25O4
C20H27O4	331.191483	1-	C20H27O4
C21H29O4	345.207133	1-	C21H29O4
C16H31O2	255.232954	1-	C16H31O2
C18H33O2	281.248604	1-	C18H33O2
C18H35O2	283.264254	1-	C18H35O2
C10H11O4	195.066282	1-	C10H11O4
C11H13O4	209.081932	1-	C11H13O4
C12H15O4	223.097583	1-	C12H15O4
C13H19O4	239.128883	1-	C13H19O4
C13H17O4	237.113233	1-	C13H17O4
C14H19O4	251.128883	1-	C14H19O4
C15H21O4	265.144533	1-	C15H21O4
C16H23O4	279.160183	1-	C16H23O4
C17H25O4	293.175833	1-	C17H25O4
C18H27O4	307.191483	1-	C18H27O4
C13H13O4	233.081932	1-	C13H13O4
C12H11O4	219.066282	1-	C12H11O4
C11H9O4	205.050632	1-	C11H9O4
C10H13O4	197.081932	1-	C10H13O4
C11H15O4	211.097583	1-	C11H15O4
C12H17O4	225.113233	1-	C12H17O4
C18H21O4	301.144533	1-	C18H21O4
C17H19O4	287.128883	1-	C17H19O4
C16H17O4	273.113233	1-	C16H17O4
C15H15O4	259.097583	1-	C15H15O4
C14H13O4	245.081932	1-	C14H13O4
C13H11O4	231.066282	1-	C13H11O4
C12H9O4	217.050632	1-	C12H9O4
C19H23O4	315.160183	1-	C19H23O4
C20H25O4	329.175833	1-	C20H25O4
C21H27O4	343.191483	1-	C21H27O4
C22H29O4	357.207133	1-	C22H29O4
C22H31O4	359.222783	1-	C22H31O4
C24H35O4	387.254083	1-	C24H35O4
C25H37O4	401.269733	1-	C25H37O4
C26H39O4	415.285383	1-	C26H39O4
C27H41O4	429.301033	1-	C27H41O4
C28H43O4	443.316683	1-	C28H43O4
C29H45O4	457.332334	1-	C29H45O4
C30H47O4	471.347984	1-	C30H47O4
C16H17O8	337.092891	1-	C16H17O8
C17H19O8	351.108541	1-	C17H19O8
C18H21O8	365.124191	1-	C18H21O8
C19H23O8	379.139841	1-	C19H23O8
C20H25O8	393.155491	1-	C20H25O8
C21H27O8	407.171141	1-	C21H27O8
C22H29O8	421.186791	1-	C22H29O8
C23H31O8	435.202442	1-	C23H31O8
C29H37O4	449.269733	1-	C29H37O4
C30H39O4	463.285383	1-	C30H39O4
C29H51O4	463.379284	1-	C29H51O4
C28H49O4	449.363634	1-	C28H49O4
C31H43O4	479.316683	1-	C31H43O4
C27H47O4	435.347984	1-	C27H47O4
C28H35O4	435.254083	1-	C28H35O4
C26H45O4	421.332334	1-	C26H45O4
C27H33O4	421.238433	1-	C27H33O4
C25H43O4	407.316683	1-	C25H43O4
C26H31O4	407.222783	1-	C26H31O4
C24H41O4	393.301033	1-	C24H41O4
C25H29O4	393.207133	1-	C25H29O4
C23H39O4	379.285383	1-	C23H39O4
C24H27O4	379.191483	1-	C24H27O4
C22H37O4	365.269733	1-	C22H37O4
C23H25O4	365.175833	1-	C23H25O4
C21H35O4	351.254083	1-	C21H35O4
C22H23O4	351.160183	1-	C22H23O4
C20H33O4	337.238433	1-	C20H33O4
C21H21O4	337.144533	1-	C21H21O4
C20H19O4	323.128883	1-	C20H19O4
C18H29O4	309.207133	1-	C18H29O4
C19H17O4	309.113233	1-	C19H17O4
C17H27O4	295.191483	1-	C17H27O4
C18H15O4	295.097583	1-	C18H15O4
C17H13O4	281.081932	1-	C17H13O4
C16H11O4	267.066282	1-	C16H11O4
C15H9O4	253.050632	1-	C15H9O4
C14H7O4	239.034982	1-	C14H7O4
C30H53O4	477.394934	1-	C30H53O4
C27H25O8	477.155491	1-	C27H25O8
C28H27O8	491.171141	1-	C28H27O8
C32H57O4	505.426234	1-	C32H57O4
C29H29O8	505.186791	1-	C29H29O8
C30H31O8	519.202442	1-	C30H31O8
C33H59O4	519.441884	1-	C33H59O4
C31H33O8	533.218092	1-	C31H33O8
C32H35O8	547.233742	1-	C32H35O8
C33H37O8	561.249392	1-	C33H37O8
C34H39O8	575.265042	1-	C34H39O8
C35H41O8	589.280692	1-	C35H41O8
C33H33O10	589.207921	1-	C33H33O10
C34H35O10	603.223571	1-	C34H35O10
C36H43O8	603.296342	1-	C36H43O8
C35H37O10	617.239221	1-	C35H37O10
C36H39O10	631.254871	1-	C36H39O10
C37H41O10	645.270521	1-	C37H41O10
C38H43O10	659.286171	1-	C38H43O10
C39H45O10	673.301821	1-	C39H45O10
C40H47O10	687.317471	1-	C40H47O10
C38H39O12	687.244700	1-	C38H39O12
C39H41O12	701.260350	1-	C39H41O12
C41H39O11	707.249786	1-	C41H39O11
C42H41O11	721.265436	1-	C42H41O11
C43H43O11	735.281086	1-	C43H43O11
C44H45O11	749.296736	1-	C44H45O11
C45H47O11	763.312386	1-	C45H47O11
C32H31O10	575.192271	1-	C32H31O10
C31H29O10	561.176621	1-	C31H29O10
C30H27O10	547.160971	1-	C30H27O10
C18H31O2	279.232954	1-	C18H31O2
C16H21O4	277.144533	1-	C16H21O4
C14H17O6	281.103062	1-	C14H17O6
C16H13O4	269.081932	1-	C16H13O4
C7H7O4	155.034982	1-	C7H7O4
C8H9O4	169.050632	1-	C8H9O4
C9H11O4	183.066282	1-	C9H11O4
C6H5O2	109.029503	1-	C6H5O2
C7H7O2	123.045153	1-	C7H7O2
C8H9O2	137.060803	1-	C8H9O2
C9H11O2	151.076453	1-	C9H11O2
C10H13O2	165.092103	1-	C10H13O2
C11H15O2	179.107753	1-	C11H15O2
C24H33O8	449.218092	1-	C24H33O8
C25H35O8	463.233742	1-	C25H35O8
C26H37O8	477.249392	1-	C26H37O8
C31H55O4	491.410584	1-	C31H55O4
C20H21O10	421.114020	1-	C20H21O10
C19H19O10	407.098370	1-	C19H19O10
C20H27O8	395.171141	1-	C20H27O8
C21H29O8	409.186791	1-	C21H29O8
C22H31O8	423.202442	1-	C22H31O8
C23H33O8	437.218092	1-	C23H33O8
C19H25O8	381.155491	1-	C19H25O8
C18H23O8	367.139841	1-	C18H23O8
C17H21O8	353.124191	1-	C17H21O8
C16H19O8	339.108541	1-	C16H19O8
C15H17O8	325.092891	1-	C15H17O8
C14H15O8	311.077241	1-	C14H15O8
C13H13O8	297.061591	1-	C13H13O8
C21H25O10	437.145321	1-	C21H25O10
C22H27O10	451.160971	1-	C22H27O10
C23H29O10	465.176621	1-	C23H29O10
C24H31O10	479.192271	1-	C24H31O10
C25H33O10	493.207921	1-	C25H33O10
C26H35O10	507.223571	1-	C26H35O10
C31H35O8	535.233742	1-	C31H35O8
C32H37O8	549.249392	1-	C32H37O8
C33H39O8	563.265042	1-	C33H39O8
C34H41O8	577.280692	1-	C34H41O8
C35H43O8	591.296342	1-	C35H43O8
C30H33O8	521.218092	1-	C30H33O8
C29H31O8	507.202442	1-	C29H31O8
C28H29O8	493.186791	1-	C28H29O8
C27H27O8	479.171141	1-	C27H27O8
C26H25O8	465.155491	1-	C26H25O8
C25H23O8	451.139841	1-	C25H23O8
C24H21O8	437.124191	1-	C24H21O8
C23H19O8	423.108541	1-	C23H19O8
C22H17O8	409.092891	1-	C22H17O8
C36H37O10	629.239221	1-	C36H37O10
C37H39O10	643.254871	1-	C37H39O10
C38H41O10	657.270521	1-	C38H41O10
C39H43O10	671.286171	1-	C39H43O10
C40H45O10	685.301821	1-	C40H45O10
C41H47O10	699.317471	1-	C41H47O10
C42H49O10	713.333121	1-	C42H49O10
C35H35O10	615.223571	1-	C35H35O10
C34H33O10	601.207921	1-	C34H33O10
C33H31O10	587.192271	1-	C33H31O10
C32H29O10	573.176621	1-	C32H29O10
C31H27O10	559.160971	1-	C31H27O10
C30H25O10	545.145321	1-	C30H25O10
C20H39O2	311.295554	1-	C20H39O2
C22H43O2	339.326854	1-	C22H43O2
C24H47O2	367.358154	1-	C24H47O2
C17H33O2	269.248604	1-	C17H33O2
C15H29O2	241.217304	1-	C15H29O2

Table S4. *Calibration mass list for (+)APPI*

Name; m/z value; charge; ion formula

C12H17	161.132477	1+	C12H17
C13H17	173.132477	1+	C13H17
C14H19	187.148127	1+	C14H19
C15H21	201.163777	1+	C15H21
C15H24	204.187252	1+	C15H24
C16H23	215.179427	1+	C16H23
C18H27	243.210727	1+	C18H27
C19H25	253.195077	1+	C19H25
C20H29	269.226377	1+	C20H29
C20H32	272.249852	1+	C20H32
C19H29O	273.221292	1+	C19H29O
C20H30O	286.229117	1+	C20H30O
C19H27O2	287.200557	1+	C19H27O2
C22H33	297.257677	1+	C22H33
C20H30O2	302.224032	1+	C20H30O2
C24H35	323.273328	1+	C24H35
C25H39	339.304628	1+	C25H39
C27H41	365.320278	1+	C27H41
C26H47O	375.362143	1+	C26H47O
C26H33O2	377.247507	1+	C26H33O2
C25H31O3	379.226771	1+	C25H31O3
C27H39O	379.299542	1+	C27H39O
C22H36O5	380.255726	1+	C22H36O5
C27H40O	380.307367	1+	C27H40O
C28H44	380.343753	1+	C28H44
C25H33O3	381.242421	1+	C25H33O3
C26H37O2	381.278807	1+	C26H37O2
C28H45	381.351578	1+	C28H45
C25H49O2	381.372707	1+	C25H49O2
C22H38O5	382.271376	1+	C22H38O5
C24H31O4	383.221686	1+	C24H31O4
C27H43O	383.330842	1+	C27H43O
C28H47	383.367228	1+	C28H47
C28H47O4	447.346886	1+	C28H47O4
C28H33O	385.252592	1+	C28H33O
C25H37O3	385.273721	1+	C25H37O3
C26H41O2	385.310107	1+	C26H41O2
C22H26O6	386.172390	1+	C22H26O6
C27H31O2	387.231857	1+	C27H31O2
C24H35O4	387.252986	1+	C24H35O4
C26H43O2	387.325757	1+	C26H43O2
C25H40O3	388.297197	1+	C25H40O3
C26H45O2	389.341407	1+	C26H45O2
C25H42O3	390.312847	1+	C25H42O3
C26H31O3	391.226771	1+	C26H31O3
C27H35O2	391.263157	1+	C27H35O2
C24H39O4	391.284286	1+	C24H39O4
C26H47O2	391.357057	1+	C26H47O2
C27H37O2	393.278807	1+	C27H37O2
C28H41O	393.315192	1+	C28H41O
C29H45	393.351578	1+	C29H45
C26H35O3	395.258071	1+	C26H35O3
C27H39O2	395.294457	1+	C27H39O2
C26H51O2	395.388357	1+	C26H51O2
C26H37O3	397.273721	1+	C26H37O3
C27H41O2	397.310107	1+	C27H41O2
C28H45O	397.346492	1+	C28H45O
C27H42O2	398.317932	1+	C27H42O2
C25H35O4	399.252986	1+	C25H35O4
C26H39O3	399.289372	1+	C26H39O3
C27H43O2	399.325757	1+	C27H43O2
C28H47O	399.362143	1+	C28H47O
C29H51	399.398528	1+	C29H51
C25H37O4	401.268636	1+	C25H37O4
C30H41	401.320278	1+	C30H41
C26H41O3	401.305022	1+	C26H41O3
C27H45O2	401.341407	1+	C27H45O2
C28H35O2	403.263157	1+	C28H35O2
C30H43	403.335928	1+	C30H43
C28H51O	403.393443	1+	C28H51O
C25H40O4	404.292111	1+	C25H40O4
C26H44O3	404.328497	1+	C26H44O3
C30H45	405.351578	1+	C30H45
C28H53O	405.409093	1+	C28H53O
C29H41O	405.315192	1+	C29H41O
C30H46	406.359403	1+	C30H46
C27H35O3	407.258071	1+	C27H35O3
C28H39O2	407.294457	1+	C28H39O2
C28H55O	407.424743	1+	C28H55O
C27H37O3	409.273721	1+	C27H37O3
C29H47O	411.362143	1+	C29H47O
C30H51	411.398528	1+	C30H51
C26H37O4	413.268636	1+	C26H37O4
C28H47O2	415.357057	1+	C28H47O2
C30H55	415.429828	1+	C30H55
C30H41O	417.315192	1+	C30H41O
C28H49O2	417.372707	1+	C28H49O2
C29H39O2	419.294457	1+	C29H39O2
C30H43O	419.330842	1+	C30H43O
C29H55O	419.424743	1+	C29H55O
C30H45O	421.346492	1+	C30H45O
C30H46O	422.354318	1+	C30H46O
C29H43O2	423.325757	1+	C29H43O2
C29H59O	423.456043	1+	C29H59O
C30H48O	424.369968	1+	C30H48O
C29H45O2	425.341407	1+	C29H45O2
C27H39O4	427.284286	1+	C27H39O4
C27H41O4	429.299936	1+	C27H41O4
C30H55O	431.424743	1+	C30H55O
C30H41O2	433.310107	1+	C30H41O2
C30H43O2	435.325757	1+	C30H43O2
C30H45O2	437.341407	1+	C30H45O2
C30H47O2	439.357057	1+	C30H47O2
C30H49O2	441.372707	1+	C30H49O2
C32H47O	447.362143	1+	C32H47O
C26H55O5	447.404401	1+	C26H55O5
C19H26O3	302.187646	1+	C19H26O3
C20H23O3	311.164171	1+	C20H23O3
C19H23O4	315.159086	1+	C19H23O4
C19H28O4	320.198211	1+	C19H28O4
C20H25O4	329.174736	1+	C20H25O4
C20H25O6	361.164565	1+	C20H25O6
C21H28O6	376.188040	1+	C21H28O6
C21H31O6	379.211515	1+	C21H31O6
C24H34O6	418.234990	1+	C24H34O6
C30H37O6	493.258465	1+	C30H37O6
C38H45O9	645.305809	1+	C38H45O9
